# Biochemical, Structural, and Conformational Characterization of a Fungal Ethylene-Forming Enzyme

**DOI:** 10.1021/acs.biochem.5c00038

**Published:** 2025-03-07

**Authors:** Shramana Chatterjee, Joel A. Rankin, Mark A. Farrugia, Simahudeen Bathir J S Rifayee, Christo Z. Christov, Jian Hu, Robert P. Hausinger

**Affiliations:** † Department of Microbiology and Molecular Genetics, Michigan State University, East Lansing, Michigan 48824, United States; ‡ Department of Chemistry, Michigan Technological University, Houghton, Michigan 49931, United States; § Department of Biochemistry and Molecular Biology, Michigan State University, East Lansing, Michigan 48824, United States; ∥ Department of Chemistry, Michigan State University, East Lansing, Michigan 48824, United States

## Abstract

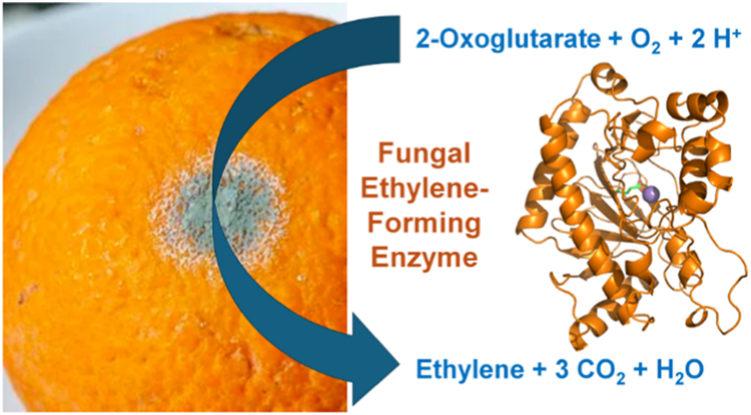

The ethylene-forming enzyme (EFE) from the fungus *Penicillium digitatum* strain Pd1 was heterologously produced in *Escherichia coli* and its properties were compared to the extensively characterized bacterial enzyme from *Pseudomonas savastanoi* strain PK2. Both enzymes catalyze four reactions: the conversion of 2-oxoglutarate (2OG) to ethylene and CO_2_, oxidative decarboxylation of 2OG coupled to l-arginine (l-Arg) hydroxylation, uncoupled oxidative decarboxylation of 2OG, and the production of 3-hydroxypropionate (3-HP) from 2OG. The strain Pd1 enzyme exhibited a greater ratio of ethylene production over l-Arg hydroxylation than the PK2 strain EFE. The uncoupled decarboxylation of 2OG and 3-HP production are minor reactions in both cases, but they occur to a greater extent using the fungal enzyme. Additional distinctions of the fungal versus bacterial enzyme are noted in the absorbance maxima and l-Arg dependence of their anaerobic electronic spectra. The structures of the Pd1 EFE apoprotein and the EFE·Mn(II)·2OG complex resembled the corresponding structures of the PK2 enzyme, but notable structural differences were observed in the computationally predicted Pd1 EFE·Fe(II)·2OG·l-Arg complex versus the PK2 EFE·Mn(II)·2OG·l-Arg crystal structure. These studies extend our biochemical understanding and represent the first structural and conformational characterization of a eukaryotic EFE.

## Introduction

Ethylene serves as a building block for the synthesis of several plastics and other organic products as well as being promoted as a transportation fuel.^1,2^ Commercial production of this gas currently involves steam cracking of fossil fuels and is the largest CO_2_-emitting process in the chemical industry.^3,4^ Therefore, there is great interest in developing alternative technology for ethylene production in a more sustainable manner using renewable sources.^5−7^ Ethylene is naturally produced in plants and by several enzymes in microorganisms.^8^ Of particular interest here is the microbial ethylene-forming enzyme (EFE), which uses 2-oxoglutarate (2OG) and arginine (l-Arg) as substrates.

**Figure 1:**
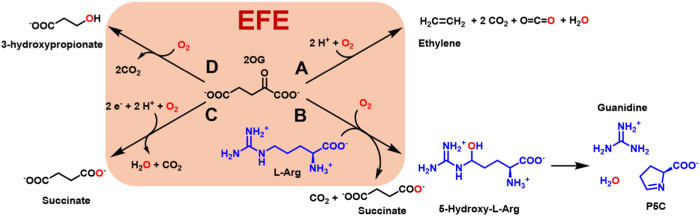
Reactions catalyzed by EFE. (A) The major reaction generates ethylene from 2OG in the presence of l-Arg. (B) Oxidative decarboxylation of 2OG drives l-Arg hydroxylation with subsequent spontaneous degradation to guanidine and P5C. (C) Uncoupled oxidative decarboxylation reaction is likely, but this reaction had not been demonstrated until now. (D) Alternative 2OG decomposition product produced in the presence of l-Arg.

EFE likely catalyzes four reactions as shown in Figure 1. Most notably, EFE uniquely converts 2OG to ethylene and CO_2_ (**reaction A**), but does so only in the presence of l-Arg.^9^ In addition, the enzyme catalyzes the oxidative decarboxylation of 2OG to produce succinate and CO_2_ coupled to the hydroxylation of l-Arg at C5 (**reaction B**).^10^ This transformation corresponds to a canonical type of reaction observed in the Fe(II)/2OG-dependent oxygenase superfamily.^11^ The hydroxy-arginine intermediate spontaneously decays to form guanidine and l-Δ^1^-pyrroline-5-carboxylate (P5C). Although not yet reported, EFE also likely catalyzes the uncoupled decarboxylation of 2OG to produce succinate and CO_2_ (**reaction C**); this reaction is widely observed in the Fe(II)/2OG oxygenases.^12^ Finally, in the presence of l-Arg, EFE produces trace amounts of 3-hydroxypropionate (3-HP) (**reaction D**),^13,14^ a precursor of a biodegradable plastic and an industrial polymer. The relative rates for the major reactions A and B, initially reported to be 2,^10^ is referred to as the partition ratio.

Most biochemical studies and all structural studies have thus far been performed with EFE from *Pseudomonas savastanoi* (formerly *P. syringae*) *pv phaseolicola* strain PK2, henceforth termed PK2 EFE.^10,15−18^ For example, the PK2 enzyme was used to demonstrate that l-Arg binding leads to significant conformational changes at the active site that facilitate ethylene formation.^16^ Mechanistic experiments and computational analyses of the ethylene-forming and l-Arg hydroxylation reactions also have centered on this enzyme and are well advanced.^13,14,19−24^ Other bacterial EFEs from *Microcoleus asticus*, *Myxococus stipitatus* DSM 14675, *Ralstonia solanacearum*, *Scytonema* sp. NIES-4073, and *Nostoc* sp. ATCC 43529 have been purified and their kinetics of ethylene formation were characterized.^25^ In contrast, EFEs from fungal sources are relatively underexplored. The enzyme was purified from *Penicillium digitatum* strain IFO 9372 and some properties were characterized; e.g., the enzyme’s activity was stated to require 2OG, l-Arg, ferrous ions, dithiothreitol, and oxygen.^26,27^ Subsequently, the genome sequence was determined for strain Pd1 of this necrotrophic fungus,^28^ which causes the common citrus postharvest disease known as green mold.

In this study, we describe the purification and extensive characterization of a mitochondrial targeting sequence-removed version of EFE from *P. digitatum* strain Pd1 (denoted Pd1 EFE) that closely corresponds to the form present in the fungus. The genome-encoded protein has an extended N-terminal sequence compared to PK2 (Figure S1); however, this extension is removed during organellar processing to the mitochondria.^29^ The overall sequence identity between the processed Pd1 and PK2 is ∼55% (188/345 residues). We compared several properties of the truncated Pd1 and PK2 versions of EFE, revealing overall similarities and several notable distinctions. Using anaerobic UV–visible spectroscopy, we reveal that the Pd1 protein exhibits changes in absorption maxima and l-Arg dependence from what was reported for PK2 EFE. We demonstrate that both versions of EFE carry out **reaction C**, as anticipated, with the Pd1 enzyme having twice the amount of uncoupled reaction as PK2 EFE. Furthermore, we establish that Pd1 EFE has a greater partition ratio (when measured as ethylene/P5C, reflecting the amount of reaction A divided by the extent of reaction B) than observed with the PK2 EFE, which has a ratio that is greater than previously reported.^10^ Using a mass spectrometry method, we show that Pd1 EFE produces more 3-HP (reflecting reaction D) than is detected for PK2 EFE. We obtained the crystal structure of Pd1 EFE apoprotein and its Mn(II)·2OG complex and show their overall structural similarity with the structures of PK2 EFE. We used computational methods to predict the structure of the Pd1 EFE·Fe(II)·2OG·l-Arg complex and to identify the O_2_ access tunnel. In combination, the structures and calculations reported here provide keen insights into the basis for the distinct properties of this fungal enzyme, the first characterized eukaryotic EFE, relative to its bacterial counterparts.

## Materials and Methods

### Chemicals

4-Bromo-*N*-methylbenzylamine (4-BNMA), tris(2-carboxyethyl)phosphine (TCEP), l-Arg, sodium phosphate monobasic, and imidazole were acquired from Sigma (St. Louis, Missouri). Kanamycin, isopropyl β-d-1-thiogalactopyranoside (IPTG), 4-(2-hydroxyethyl)-1-piperazineethanesulfonic acid (HEPES), and nickel-nitrilotriacetic acid (Ni-NTA) agarose beads were purchased from GoldBio (St. Louis, Missouri). 2OG was from Fluka, EDTA was from Invitrogen, 3-HP (sodium salt) was from Cayman Chemical, and all other chemicals were of reagent grade or better.

### Plasmid Construction

Using the *P. digitatum* strain Pd1 genome,^28^ we had synthesized (Integrated DNA Technologies, Inc.) a codon-optimized version of the gene that directly connects the four predicted exons encoding EFE preceded by a His_6_ tag and a tobacco etch virus (TEV) protease cleavage site (Glu-Asn-Leu-Tyr-Phe-Gln-*-Ser, with * denoting the cleavage point) for expression in *Escherichia coli*. This construct yielded mainly insoluble protein because the initially predicted start codon had been mis-identified and the protein was missing its first 49 residues, some of which are removed during organellar targeting in the fungus.^29^ The initial construct was modified to provide a soluble TEV-cleaved protein that started with Ser from the TEV cleavage site, a His-Met linker, followed by Leu37 and the remainder of EFE using the polymerase chain reaction (PCR) with Q5 high-fidelity DNA polymerase (New England Bio Laboratories) and the following primers (Integrated DNA Technologies, Inc.): forward, 5′-GCCGTAGAATTCTTGACGACTACTACAGCA-3′, and reverse, 5′-CGGCATGGATCCTGAAAGTAGGCGAAAGC-3′. The PCR product was digested with NdeI and BamHI restriction enzymes, and the fragment was ligated into a modified pET28a(+) vector (carrying kanamycin resistance) with codons for an N-terminal His_6_ tag and a TEV cleavage site. After the plasmid sequence was verified at the RTSF Genomics Core of Michigan State University, it was transformed into *E. coli* BL21 Gold (DE3) cells (Agilent Technologies). Using Vazyme Clone Express kit C112, mutagenesis of this plasmid was performed to generate a construct encoding the N380C variant form of Pd1 EFE.

### Enzyme Purification

Unless otherwise noted, all buffers were prepared at room temperature with the pH adjusted using either NaOH or HCl. Terrific broth medium supplemented with 50 μg/mL kanamycin (1 L in 2.8 L Fernbach flasks) was inoculated (1%) with a fresh culture and grown overnight at 37 °C with shaking at 225 rpm until reaching an OD_600_ of 0.8–1.2. The temperature of the culture was lowered to 20 °C, the cells were induced with IPTG (final concentration of 0.2 mM), and growth continued overnight. The next day, the culture was harvested by centrifugation at 7000*g* and 4 °C for 15–20 min. The cells were stored at −80 °C until further use. The same culture method was used for production of the N380C variant of Pd1 EFE. The cells producing the variant enzyme grew more slowly than those synthesizing the nonvariant EFE.

The cell pellets were thawed, resuspended in buffer A [50 mM NaH_2_PO_4_ (pH 8.0), 100 mM NaCl, and 10 mM imidazole], and supplemented with 1 mM phenylmethylsulfonyl fluoride (from a 100 mM stock in 100% ethanol) and 1 U/mL Benzonase (EMD Millipore). The cell suspensions were lysed by using a French pressure cell at 16,000 psi at 4 °C. Lysates were clarified by centrifugation (45 min at 100,000*g*) at 4 °C. The clarified lysates were applied to a Ni-NTA agarose column, unbound proteins were eluted with 10 column volumes of buffer A, and the protein of interest was eluted with ∼5–10 column volumes of buffer B (buffer A with 300 mM imidazole).

The Ni-NTA fractions containing the desired Pd1 EFE protein were concentrated, and the buffer was exchanged for 50 mM NaH_2_PO_4_ (pH 8.0) containing 100 mM NaCl and 10 mM imidazole by using a 10 kDa molecular weight cutoff Amicon Ultra-15 centrifugal filter unit (EMD Millipore). The His_6_ tag was removed by incubation with His_7_-TEV238Δ protease^30^ for 16–18 h at 4 °C, and the EFE/TEV protease mixture was applied to the Ni-NTA column that had been equilibrated with buffer A. The flow-through fractions and ∼7–10 column volumes of buffer A wash were collected, concentrated to 2.5 mL, and buffer exchanged into 25 mM HEPES buffer (pH 8.0) containing 1 mM EDTA and 1 mM dithiothreitol (DTT) using a PD-10 column (Cytiva). Prior to performing any assays, EDTA was removed from the protein of interest using a PD-10 column. For long-term storage, the eluted fractions were concentrated, glycerol was added to a final concentration of 5%, the samples were flash-frozen in liquid nitrogen, and the enzyme was placed at −80 °C until further use. For comparative studies, we purified the strain PK2 EFE and its A198V variant^16^ using a previously described protocol.^15^

The homogeneity of the EFE samples was assessed by sodium dodecyl sulfate-polyacrylamide gel electrophoresis (SDS-PAGE).^31^ To more accurately determine the subunit size of Pd1 EFE, protein samples (10 μM) were injected onto a cyano-chemistry HPLC column that was equilibrated in 0.1% formic acid and eluted with an increasing gradient of acetonitrile. The fractions were analyzed by electrospray ionization-mass spectrometer (ESI-MS) using a XEVO G2-XS instrument in positive ionization mode. The protein masses were derived from the MS data using MaxEnt1 (Waters Corp).^32^ The mass of the native enzyme was estimated by size exclusion chromatography (SEC) on a Superdex 200 Increase 10/300 GL (Cytiva) column equilibrated with 25 mM HEPES, pH 8.0, containing 1 mM TCEP. The SEC fractions with significant 280 nm absorbance were analyzed by injecting (8 μL) into the ESI-MS to determine the subunit size.

### Metal Analysis

PK2 and Pd1 versions of EFE were purified as described above and, after removing the His_6_ tag with TEV protease, they were (1) maintained in 50 mM NaH_2_PO_4_ (pH 8.0) and 100 mM NaCl buffer, (2) exchanged into 25 mM HEPES (pH 8.0) buffer, or (3) exchanged into 25 mM HEPES (pH 8.0) buffer containing 1 mM EDTA and 1 mM DTT followed by EDTA removal using a PD-10 column. The concentrations of Pd1 EFE for the three conditions were 812, 997, and 950 μM, and those of PK2 EFE were 2900, 3109, and 2433 μM, respectively. EFE samples (25–150 μL) were mixed with 100 μL of 70% nitric acid and digested for 1 h at 100 °C, diluted to 5 mL with water, and examined using an Agilent 8900 Triple Quadrupole ICP-MS at the MSU Quantitative Bio Element Analysis and Mapping (QBEAM) Center to determine the metal contents.

### Anaerobic UV–Visible Spectroscopy

Stock solutions (100 mM) of 2OG and l-Arg were prepared in 25 mM HEPES buffer (pH adjusted to 7.5) in serum vials sealed with butyl rubber stoppers and made anaerobic by several rounds of vacuum degassing and flushing with argon via a vacuum manifold. After degassing, sodium dithionite was added to a final concentration of 2 mM from a 100 mM stock solution. Ferrous ammonium sulfate stock solutions (100 mM) were prepared by several rounds of degassing and flushing with argon inside a sealed serum vial. The Fe(NH_4_)_2_(SO_4_)_2_ salt was dissolved in the desired volume of 25 mM HEPES buffer (pH 7.5) containing 2 mM sodium dithionite. The Pd1 EFE sample was made anaerobic by multiple rounds of gentle degassing and flushing with argon on ice, then adjusted to contain 2 mM sodium dithionite. All equilibrium spectroscopy studies used a 1 cm path length, 2 mL quartz cuvette fitted with a stopper and purged with argon. Samples of Pd1 EFE [0.37 mM subunit in 25 mM HEPES buffer (pH 7.5)] were transferred into the cuvette using a gastight syringe (Hamilton) that had been flushed with 2 mM dithionite buffer. Difference spectra were recorded for Pd1 EFE samples to which anaerobic aliquots (10 μL) of Fe(NH_4_)_2_(SO_4_)_2_, 2OG, and l-Arg had been added, blanking against the enzyme and dithionite mixture.

### Enzyme Assays

Enzyme assays were performed at room temperature (22 ± 1 °C) unless noted otherwise in 10 mm × 16 mm tubes (BD Vacutainer Serum). Aliquots of EFE (using varied amounts as noted in the text or figure legends) were incubated in 2 mL of 25 mM HEPES buffer (pH 7.5) containing the indicated concentrations of 2OG, l-Arg, Fe(NH_4_)_2_(SO_4_)_2_, and l-ascorbic acid. The reactions were vortexed and then terminated at designated time points by adding 0.1 mL of 3.6 M HCl unless mentioned otherwise. Ethylene formation was measured by withdrawing 0.25 mL of the headspace with a Hamilton gastight syringe and injecting it into a gas chromatograph (Shimadzu GC-8A) equipped with a flame ionization detector and a Porapak N-packed column (80/100 mesh, 2 m × 1/8 inch). The instrument was calibrated using known concentrations of ethylene (SCOTTY Analyzed Gases, 99.5%).

The concentrations of P5C were determined by either of two approaches. In one case, an aliquot (1 mL) of the reaction solution was mixed with 0.2 mL of 10 mM 2-aminobenzaldehyde in 40% ethanol, incubated at 37 °C for 20 min to develop the yellow adduct, and the absorbance was measured at 440 nm, using an extinction coefficient of 2.58 mM^–1^ cm^–1^.^33^ Alternatively, the reactions were terminated with 0.1 mL of 3.6 M HCl, and 1 mL samples were derivatized by adding 0.2 mL of 2% ninhydrin in water. The mixtures were heated to 100 °C for ∼30 min, cooled, and centrifuged at 3234*g* for 15–30 min at 4 °C. After decanting the supernatant, the reddish-brown sediment was resuspended in ethanol (0.5 mL), vortexed to extract the P5C-ninhydrin chromogen (a pinkish color), and transformed to a bluish color by adding 0.5 mL of 50 mM Tris-HCl buffer (pH 8.0). Following centrifugation at 2500*g* for 10 min, the absorbance at 620 nm was measured and the concentration of P5C was calculated based on the established molar extinction coefficient of 1.96 mM^–1^ cm^–1^ for the P5C–ninhydrin adduct.^34^

Succinate and 2OG concentrations^35^ were determined in samples and standards that were heat treated (60 °C for 15–20 min) using a Waters TQ-S triple quadrupole MS/MS controlled by MassLynx 4.2 software (Waters, Milford, MA). MS/MS data were evaluated with TargetLynx 4.2 software (Waters). The autosampler injected 5 μL of sample from a compartment maintained at 10 °C. The chromatographic separation of the analyte was performed at 50 °C on a Waters Acquity BEH C18 (2.1 × 50 mm) analytical column using 10 mM tributylamine and 15 mM acetic acid in 97% water and 3% methanol (MS grade) for eluent A and methanol (MS grade) for eluent B. Gradient elution at a flow of 0.3 mL/min was performed by changing % B as follows: 0.0–1.0 min: 10%; 1.0–3.0 min: 10 to 20%; 3.0–6.0 min: 20 to 65%; 6.0–6.5 min: 65 to 95%; 6.5–8.0 min: 95%, 8.01- 10.0 min: 10%. All analytes were measured in negative electrospray ion mode using dwell times of 44 ms. Optimized MS/MS settings are summarized in Table S1. The concentrations of analytes were determined by comparison to standard curves. Notably, this assay for succinate is distinct from the HPLC method with detection by absorbance or refractive index as used previously.^15^

Quantification of the amount of 2OG that was consumed during the reaction utilized an *o*-phenylenediamine (OPDA) assay. Aliquots (250 μL) of reaction mixtures were incubated for selected time periods, quenched by the addition of 1 mL 0.5 mg/mL OPDA (Sigma-Aldrich) stock solution (dissolved in 1 M phosphoric acid, pH 2, containing 0.25% (v/v) β-mercaptoethanol), and the samples were heated for 3 min at 100 °C. The absorbance at 333 nm was monitored to determine 2OG consumption by comparison to standard curves.

The production of 3-HP^36^ was quantified in reaction mixtures and standards (100 μL) that were quenched with three volumes of acetonitrile or by adding 50 μL of methanol and 100 μL of dried pyridine. The samples were shaken in a rocker for 20 min followed by addition and mixing of 30 μL 1-ethyl-3-(3-dimethyaminopropyl)carbodiimide (EDC) solution (13.6 mg mL^–1^) in methanol-dried pyridine (20:80 v/v) and 50 μL of 4-BNMA solution (4.8 mM in dried pyridine). The tubes were maintained at 72 °C for 45 min or until the organic solvents evaporated, then the samples were dried at 72 °C under a stream of N_2_. The samples were dissolved in 150 μL of a solution containing 2 parts of 0.1% formic acid in water and one part of acetonitrile along with 350 μL of ethyl acetate. After mixing in a rocker for 20 min, the samples were centrifuged at 2500*g* for 10 min and the supernatants were transferred to new Eppendorf tubes. After drying under a stream of N_2_, the samples were reconstituted into 100 μL of water containing 0.1% formic acid and methanol (1:1 v/v). The analyte was analyzed by ESI-MS using a XEVO G2-XS instrument in positive ionization mode. MassLynx 4.2 software (Waters, Milford, MA) was used for data acquisition and analysis. The autosampler injected 10 μL of sample maintained at a compartment temperature of 10 °C. Analytes were injected onto a Waters Acquity premier BEH C18 (2.1 mm × 100 mm) column that was equilibrated in 10 mM ammonium formate in water, pH 2.8, at 40 °C and eluted with an increasing gradient of acetonitrile at a flow rate of 0.3 mL/min. The total run time per injection was 10 min. The analyte retention time and *m*/*z* of selected reaction monitoring (SRM) transitions are shown in Table S2 with examples of the derivatized structures for 3-HP, succinic acid, and 2-OG.

To obtain the kinetic constants for the EFE-catalyzed reactions of 2OG and l-Arg, the enzyme samples (125–200 nM EFE in 25 mM HEPES buffer, pH 7.5) were added to 2 mL assay mixtures containing 0.2 mM Fe(NH_4_)_2_(SO_4_)_2_, 0.4 mM l-ascorbic acid, and varying the concentrations of either 2OG or l-Arg; i.e., the concentration of one substrate was kept constant at 500 μM while varying the concentration of the other substrate (0–700 μM). The reactions were terminated at designated time points and products were quantified as described above. The initial velocity data were fitted by nonlinear regression analysis using the Michaelis–Menten equation in Origin 8.0.

### Crystallization and Data Analysis

For crystallization, TEV-cleaved Pd1 EFE was further purified by SEC using a Superdex HiLoad 16/600 75 prep grade column (GE Healthcare Life Sciences). The column was equilibrated with 25 mM HEPES, pH 8.0, containing 100 mM NaCl and 1 mM TCEP. The Pd1 EFE was concentrated and buffer exchanged into 25 mM HEPES buffer, pH 8.0, supplemented with 1 mM TCEP, and concentrated to ∼40 mg/mL using an Amicon ultracentrifugation unit (molecular-weight cutoff 10,000 Da). Crystallization was performed in 96 well plates by the sitting drop vapor diffusion technique and using the mosquito crystallization robot (TTP Labtech). Initial crystallization conditions were explored using the Index HT (Hampton Research), Crystal Screen HT (Hampton Research), Wizard 1&2 (Rigaku Reagents), and Wizard 3&4 (Rigaku Reagents). Crystals grew in a single condition containing 10% PEG 8000, 100 mM imidazole-HCl (pH 8.0), and 200 mM calcium acetate at 4 °C. The crystals of Pd1 EFE apoprotein were retrieved using a nylon loop and soaked overnight in reservoir solution supplemented with 1 mM MnCl_2_ and 2OG. Crystals were soaked in 25% ethylene glycol and 75% reservoir solution (containing MnCl_2_ and 2OG as applicable) before flash freezing in liquid nitrogen.

X-ray diffraction data were collected at the Advanced Photon Source LS-CAT beamline 21-ID-D. For details, see Table S3. Data sets were indexed and integrated with iMosflm^37^ and merging and scaling were done using Aimless.^38^ Molecular replacement and refinement were done in Phenix^39^ with model building in COOT.^40^ Initially, a model of Pd1 EFE was created by AlphaFold^41^ and used in molecular replacement to solve the structure. Data sets were deposited to the protein data bank (PDB) with IDs 9EIR and 9EIS. Structural features are depicted using PyMOL software (Shrödinger).

### System Preparation for Computational Analyses

Starting with the coordinates for Pd1 EFE·Mn(II)·2OG (chain A), the position of 2OG was reconfigured to that for 2OG in the PK2 EFE·Mn(II)·2OG·l-Arg structure (5V2Y), the l-Arg conformation was obtained from the PK2 structure by overlaying and was positioned to interact with Glu141, Thr143 and Arg336, and Mn(II) was replaced by Fe(II). The position *trans* to His251 was modified to dioxygen to obtain the Fe(III)-superoxo structure. Molecular dynamics (MD) simulations were carried out at this Fe(III)·OO^•–^ state for rigorous comparison with the PK2 EFE dynamics published in our earlier work.^19^ Hydrogen atoms were added to the protein residues using Amber routines according to their protonation states. The parameters for nonstandard residues, including 2OG, l-Arg, and dioxygen, were obtained using a Generalized Amber Force Field (GAFF)^42^ implemented in an antechamber module of Amber 20.^43^ The metal center parameters were obtained for the high spin Fe(III) (*S* = 2, and *M* = 5) state coordinated to 2OG, dioxygen, His251, Asp253, and His327 using Metal Center Parameter Builder (MCPB v3.0).^44^ The ff14SB^45^ force field was used to model the rest of the protein. The system was then solvated and neutralized with Na^+^ counterions to obtain the initial structure of Pd1 EFE·Fe(III)·OO^•–^·2OG·l-Arg.

### MD Simulations

The solvated system was minimized in two steps—the first minimization involved a restraint of 100 kcal/mol on the solute molecule, and the second minimization was carried out without any restraints. The minimization steps involved 5000 steps of the steepest descent method and the remaining 5000 steps of the conjugate gradient method. The system was heated from 0 to 300 K as an NVT ensemble using a Langevin thermostat with 1 ps^–1^ collision frequency for 250 ps.^46^ The solute molecules were restrained during heating with a harmonic potential of 50 kcal/mol. After heating, the system underwent a 1 ns simulation with a weak restraint on the solute to achieve uniform density. Before equilibration, the system underwent a 300 ns simulation with a small restraint between the Fe and the C5 carbon of l-Arg to obtain a better orientation of the l-Arg substrate. After the restraint dynamics, equilibration was carried out for 3 ns at constant pressure. Following equilibration, the production simulations were carried out for 1 μs with a time step of 2 fs. The particle mesh Ewald method^47^ was used to compute long-range interactions with a 10 Å cutoff. The simulations were implemented in the GPU-accelerated version of Amber 20 (Amber-PMEMD).^43^ Hydrogen bonding analyses were carried out using the CPPTRAJ module of Amber 20.^48^ Dynamic Cross Correlation analysis (DCCA) and Principal Component Analysis (PCA) were conducted on the Cα atoms of the protein in the equilibrated portion of the MD trajectory using the Bio3D module implemented in the R software package.^49^ The potential dioxygen transport channels were manually inspected from the equilibrated portion of the MD simulations and characterized using CAVER 3.0.^50^ The dioxygen was removed from the system for tunnel calculations.

## Results and Discussion

### Purification and Initial Properties of PK2 and Pd1 EFEs

We overexpressed in *E. coli* a codon-optimized gene for EFE from *P. digitatum* strain Pd1 that was preceded by a His_6_ tag, a TEV cleavage site, and a His-Met linker, then starting at Leu37 (i.e., missing its putative mitochondrial targeting signal, which is unstructured according to the structural model predicted by AlphaFold).^41^ The His_6_-tagged protein was synthesized in cells grown at 20 °C, purified by immobilized metal affinity chromatography, treated with TEV protease to cleave the tag, and then isolated by re-chromatography using the affinity resin. Residues in this protein retain the numbering of the nontruncated protein initially synthesized by the fungus. The sample was treated with DTT and EDTA, providing essentially homogeneous apoenzyme after removal of the reductant and chelator as shown by SDS-PAGE (Figure S2A). ESI-MS confirmed the presence of a species with *m*/*z* of 41,971 (Figure S2B), corresponding to the theoretical mass of 41,971 Da (calculated using https://web.expasy.org/protparam/ with an input of Ser-His-Met and the truncated EFE starting at Leu37 of UniprotKB entry A0A7T7BQH3). The results from size exclusion chromatography demonstrated that the apoprotein form of the native enzyme was monomeric (Figure S2C,D), as was previously reported for the holoenzyme from *P. digitatum* strain IFO 9372.^27^ For comparison, PK2 EFE was purified from recombinant *E. coli* cells as previously described.^15^

After purification by Ni-NTA chromatography, PK2 EFE was blue in color, whereas Pd1 EFE was yellow (Figure S3A,B). The visible spectrum of the PK2 enzyme exhibited a broad peak with a maximal absorption at ∼620 nm, whereas the Pd1 EFE spectrum had a gradually increasing absorbance with decreasing wavelength (Figure S3C). After cleavage of His_6_ tags, the metal contents for each purified sample were analyzed by ICP-MS (Table S4). Whereas PK2 EFE possessed 0.85 or 0.86 Ni/subunit in phosphate or HEPES buffers, the Pd1 EFE contained only 0.32 or 0.23 Ni/subunit for these conditions, and both proteins had <0.06 Fe/subunit. We attribute the blue color of PK2 EFE to *d*-*d* electronic transitions associated with the near stoichiometric amounts of Ni bound to the protein by nitrogen-containing ligands, whereas the yellow color of Pd1 EFE results from the low levels of Ni, which could possibly bind in a distinct manner, and small amounts of bound ferric ions. After treatment with 1 mM DTT and 1 mM EDTA (in HEPES buffer at pH 8.0) the colors disappeared, and the metal contents dropped to 0.026 and 0.015 Ni/subunit for both the PK2 and Pd1 proteins. All subsequent studies utilized the apoproteins of PK2 and Pd1 EFEs after removal of the thiol and chelator.

### Anaerobic Difference Spectra

**Figure 2:**
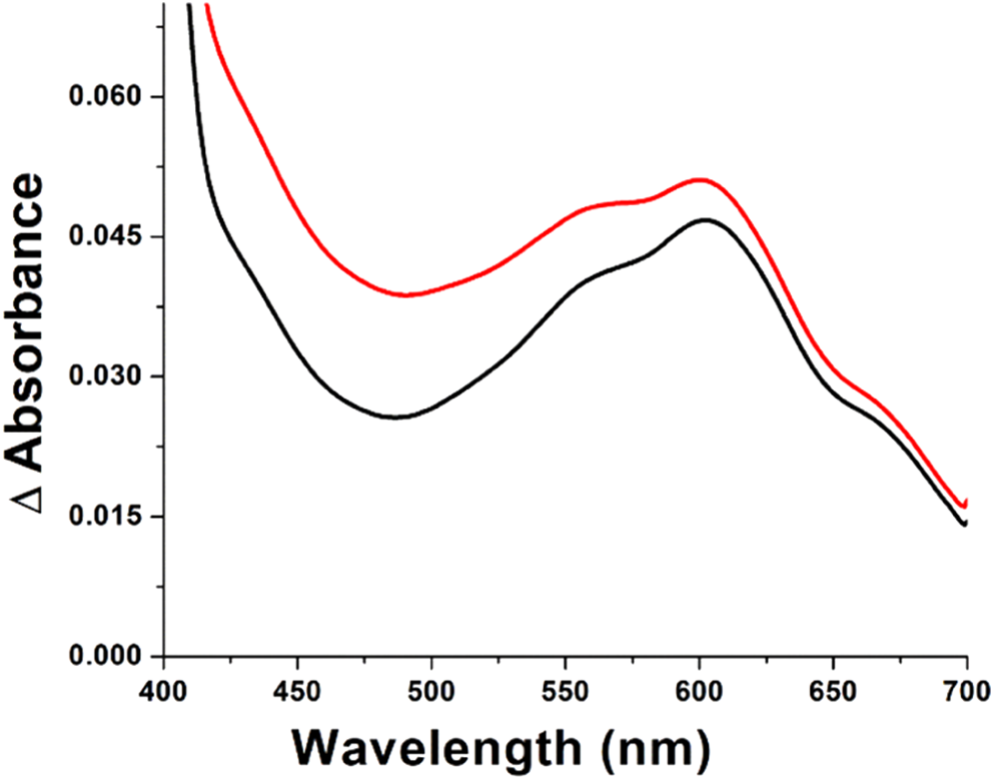
Difference spectra of Pd1 EFE·Fe(II)·2OG (black) and Pd1 EFE·Fe(II)·2OG·l-Arg (red) complexes, with the spectrum of the Pd1 EFE·Fe(II) solution taken as blank. The anaerobic samples contained 362 μM Pd1 EFE, 1 mM sodium dithionite, 1 mM ferrous ammonium sulfate, 1 mM 2OG, and (when present) 1 mM l-Arg. All the components were present in 25 mM HEPES buffer, pH 7.5. The pH of l-Arg and 2OG solutions were adjusted to 7.5 before degassing.

UV–visible difference spectroscopy was used to investigate the binding of Fe(II) and 2OG to Pd1 EFE under anaerobic conditions (Figure 2). The addition of 2OG to an anaerobic solution of Pd1 EFE containing Fe(II) gave rise to three electronic transitions with λ_max_ ∼ 565 nm, ∼ 600 nm, and ∼670 nm corresponding to extinction coefficients of ∼151, 165, 90 M^–1^ cm^–1^ (black trace). These metal-to-ligand charge-transfer (MLCT) transitions are associated with *z*^2^ → π*, *x*^2^–*y*^2^ → π*, and *yz* → π* electronic transitions where the π* energy level shifts depending on the dihedral angle between the carbonyl and carboxyl groups of the 2-oxo acid.^51^ The extinction coefficients slightly increase after addition of l-Arg with values of ∼172, 178, and 95 M^–1^ cm^–1^, respectively, for the above-mentioned λ_max_ values (red trace). These results differ significantly from what was reported previously for the PK2 enzyme.^15^ In that case, the three transitions were indistinct, and the 2OG-induced difference spectrum had a single λ_max_ of ∼515 nm with an extinction coefficient of ∼114 M^–1^ cm^–1^, whereas the addition of l-Arg led to a slight shift in λ_max_ to ∼510 nm along with a dramatic increase in extinction coefficient to 314 M^–1^ cm^–1^. The PK2 EFE results were interpreted in terms of conformational changes in the crystal structures that showed an initial mixture of monodentate and bidentate 2OG–metal interactions shifting to exclusively bidentate binding upon the binding of l-Arg.^16^ The significant l-Arg dependence of the spectral intensity was not present in the Pd1 EFE, suggesting an alternative binding mode of l-Arg in the fungal protein.

### Production of Ethylene, P5C, Succinate, and 3-HP by Pd1 EFE

**Figure 3:**
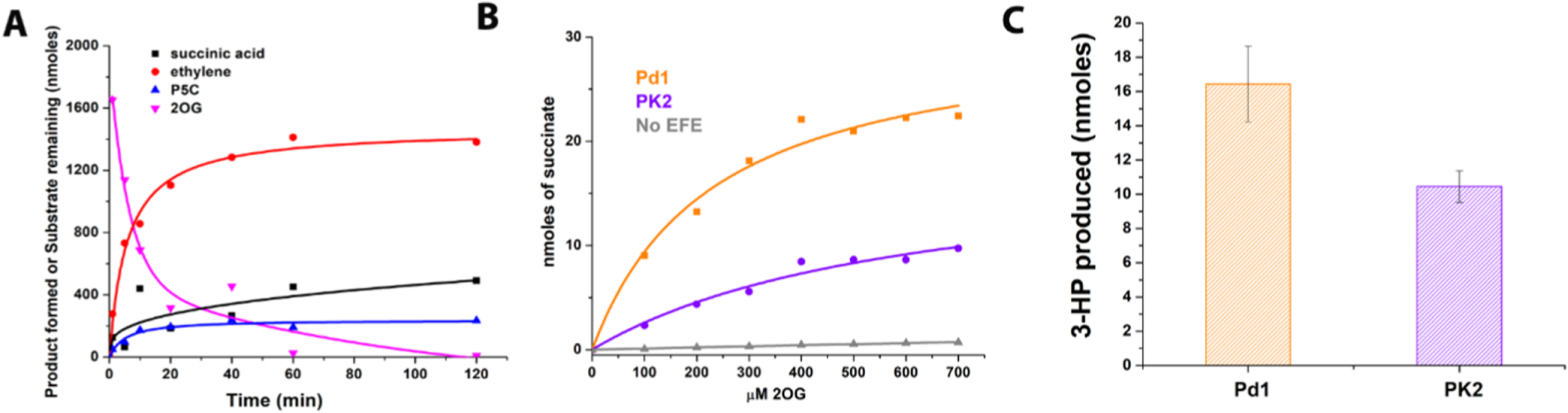
Production of ethylene, P5C, uncoupled formation of succinate, and 3-HP by EFEs. (A) Time-dependent conversion of substrate to products by Pd1 EFE. 60 μM Pd1 EFE was incubated with 6.67 mM 2OG, 6.67 mM l-Arg, 0.4 mM Fe(NH_4_)_2_(SO_4_)_2_, and 0.8 mM l-ascorbate in 0.3 mL of reaction buffer. At the times indicated the reactions were quenched with 0.9 mL of acetonitrile. Ethylene production (red) was assessed by GC. The amount of remaining 2OG (magenta) and the levels of succinate (black) were quantified by MS after derivatization with 4-BNMA, while the amount of P5C (blue) produced was measured by reaction with 2-aminobenzaldehyde. (B) Succinate production as a function of 2OG concentration by Pd1 (orange square symbols) and PK2 (purple circle symbols) versions of EFE (200 nM) in reactions free of l-Arg. The samples were incubated for 80 min at 22 °C in 25 mM HEPES buffer (pH 7.5) with 0.2 mM Fe(NH_4_)_2_(SO_4_)_2_, and 0.4 mM l-ascorbate with varying concentration of 2OG then quenched before monitoring the amount succinate formed. The results were compared to a no-enzyme control (gray triangles). (C) 3-HP production for Pd1 (orange) and PK2 (purple) versions of EFE. Enzyme samples were incubated for 120 min at 22 °C (room temperature) in 0.3 mL of 25 mM HEPES buffer (pH 7.5) with 6.67 mM 2OG, 6.67 mM l-Arg, 0.4 mM Fe(NH_4_)_2_(SO_4_)_2_, and 0.8 mM l-ascorbate. The reactions were quenched by addition of 0.9 mL of acetonitrile, then 100 μL of the quenched reaction mixture was derivatized with 4-BNMA and subjected to MS analysis. For panels A and B, nonlinear fitting was performed using Origin 8 software.

Pd1 EFE converted 2OG and l-Arg to ethylene, succinate, and P5C (Figure 3A). The final concentration of ethylene formation was approximately seven times the final concentration of P5C, which is much greater than the partition ratio of 2 initially reported for PK2 EFE.^10^ Ethylene and P5C were formed via **reactions A and B** of Figure 1. Succinate also is produced by **reaction B**, but it is formed in greater amounts than P5C consistent with some uncoupled 2OG oxidation via **reaction C**. We directly demonstrated that such an uncoupled reaction occurs in both Pd1 and PK2 versions of EFE by monitoring succinate production in the absence of l-Arg (Figure 3B). Pd1 EFE generated more than twice the amount of succinate than did PK2 EFE when the samples lacked l-Arg, whereas the levels for nonenzymatic conversion of 2OG to succinate were negligible. In the presence of l-Arg the concentrations of P5C and succinate were comparable for reactions using either the Pd1 or PK2 versions of EFE, even when the temperature was varied from 25 to 37 °C or if the pH was varied from 6.5 to 8.0 (Table S5). Small amounts of 3-HP (**reaction D** in Figure 1) were produced by both versions of EFE, with the Pd1 enzyme producing twice that of PK2 EFE (Figure 3C).

We extended our analysis of 3-HP production by examining the A198V variant of PK2 EFE. Other investigators had shown that the A198L variant of PK2 EFE exhibits greatly reduced production of ethylene while generating 0.435 3-HP per 2OG consumed,^14^ so we tested whether our previously described A198V variant^16^ also produces prodigious quantities of this side product. Indeed, this variant of PK2 EFE converted most 2OG to 3-HP (Figure S4), while using a small amount of 2OG to hydroxylate l-Arg and generating only trace levels of ethylene.^16^

### Pd1 EFE Kinetics

The Pd1 enzyme exhibited Michaelis–Menten kinetics when examining the production of ethylene or the generation of P5C as a function of either 2OG or l-Arg concentration (Figure S5). The calculated kinetic parameters are shown in Table 1 using the ninhydrin assay for quantifying P5C, with values for the PK2 enzyme also tabulated for comparison. The ethylene production *k*_cat_ values of Pd1 and PK2 EFEs are very close to each other, whereas the corresponding *K*_m_ values of Pd1 EFE are several fold greater than the former PK2 EFE values; thus, the catalytic efficiencies of Pd1 EFE for ethylene generation using these two substrates are 46 and 17% of the PK2 protein values.^15^ Other investigators reported the ethylene production kinetic parameters for the PK2 EFE and five other purified bacterial EFEs.^25^ They reported values of 22–54 min^–1^ for *k*_cat_ (2OG), 23–55 min^–1^ for *k*_cat_ (l-Arg), 20–37 μM for *K*_m_ (2OG), and 16–46 μM for *K*_m_ (l-Arg) using the bacterial enzymes, similar to what we had reported for PK2 EFE. Overall, the kinetics parameters of Pd1 EFE are similar to those for previously characterized members of this enzyme family.

**Table 1:** Kinetic Parameters for Pd1 EFE Compared to Those Previously Reported for PK2 EFE

identity of EFE	product assayed	substrate varied	*k*_cat_ (min^–1^)	*K*_m_ (μM)	*k*_cat_/*K*_m_ (μM^–1^ min^–1^)
**Pd1**	ethylene	2OG	32 ± 2	126 ± 24	0.25 ± 0.06
**PK2**	ethylene	2OG	31 ± 3	57 ± 4	0.55 ± 0.09
**Pd1**	ethylene	l-Arg	25 ± 2	162 ± 14	0.15 ± 0.02
**PK2**	ethylene	l-Arg	32 ± 1	37 ± 2	0.87 ± 0.07
**Pd1**	P5C	2OG	7.2 ± 0.2	167 ± 48	0.043 ± 0.014
**PK2**	P5C	2OG	ND^a^	ND	ND
**Pd1**	P5C	l-Arg	3.4 ± 0.3	128 ± 34	0.027 ± 0.009
**PK2**	P5C	l-Arg	0.73 ± 0.08	50 ± 7	0.015 ± 0.004

a ND, not determined.

### Comparison of the Partition Ratio for Pd1 and PK2 EFEs

**Figure 4:**
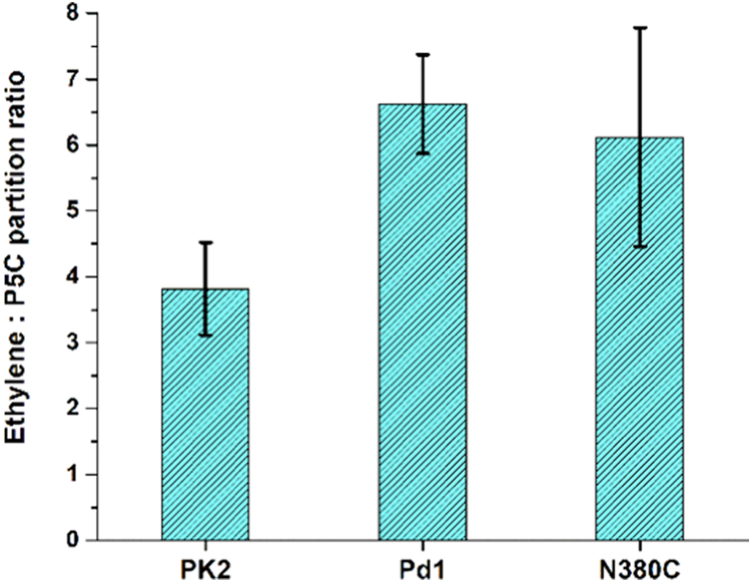
Analysis of the partition ratios for PK2, Pd1, and the N380C variant of Pd1 EFEs. The enzyme samples (250 nM) were incubated for 80 min at room temperature in 25 mM HEPES buffer (pH 7.5) with 1 mM 2OG, 1 mM l-Arg, 0.4 mM Fe(NH_4_)_2_(SO_4_)_2_, and 1 mM l-ascorbate before monitoring the amount of ethylene and P5C (using the 2-aminobenzaldehyde assay) that were formed. P values from Student’s *t*-test are 0.0001, 0.02, and 0.5 for comparing the PK2 and Pd1 enzymes, PK2 and N380C Pd1 EFEs, and the Pd1 and N380C Pd1 samples, respectively.

Prior studies of the PK2 version of EFE reported ethylene/succinate partition ratios ranging from 1 to 3,^10,14,18,21^ suggesting that the value is reaction condition dependent. Because succinate also may be produced by the uncoupled oxidative decarboxylation of 2OG (Figure 1, **reaction C**) as we demonstrated above, we chose to monitor the partition ratio of the ethylene forming reaction versus the l-Arg hydroxylation reaction by determining the ethylene/P5C ratio. Indeed, the initially reported^10^ ethylene/succinate ratio of 2 likely underestimated the true partitioning between Figure 1**A/B** as hinted by our earlier studies with the PK2 EFE enzyme.^15^ The results from Figure 3A for Pd1 EFE suggest that this enzyme may have a significantly larger partition ratio. To more accurately measure this value, we compared the ethylene/P5C partition ratios for PK2 EFE and Pd1 EFE at the same time and using identical conditions (Figure 4). The PK2 EFE exhibited a ratio of 3.8 ± 0.7, whereas the Pd1 EFE provided a ratio of 6.6 ± 0.7.

### Structure of a Fungal EFE

We obtained crystal structures for the Pd1 EFE apoprotein and the enzyme complex with both manganese and 2OG (Table S3). We illustrate a representative electron density map for a portion of the backbone and side chains (residues 64 to 87 of chain A) of the Pd1 EFE apoprotein and provide an omit map of Mn(II) in chain C (Figure S6). Mn(II) is a surrogate of Fe(II) that does not react with oxygen; although the Mn-containing enzyme is inactive, this substituted version has been used for obtaining structures of many Fe(II)/2OG oxygenases including PK2 EFE.^16^ An AlphaFold model^41^ was used as a template for molecular replacement to solve the Pd1 EFE apoprotein crystal structure at a resolution of 3.5 Å. The Pd1 EFE crystals have a different morphology but the same space group (*P*2_1_2_1_2_1_) as the PK2 EFE.^16^ Although Pd1 EFE is predominantly a monomer in solution, the asymmetric unit contains four molecules (chains A, B, C, and D) with the overall Cα RMSD for the apoprotein structure ranging from 0.19 to 0.32 Å (Table S6). We were unable to model several disordered regions of the apoprotein (chain A, residues 140-144, 237-249, 274-295; chain B, residues 139-147, 237-251, 274-294; chain C, residues 236-251, 277-295; and chain D, residues 139-150, 238-244, and 275-294).

**Figure 5:**
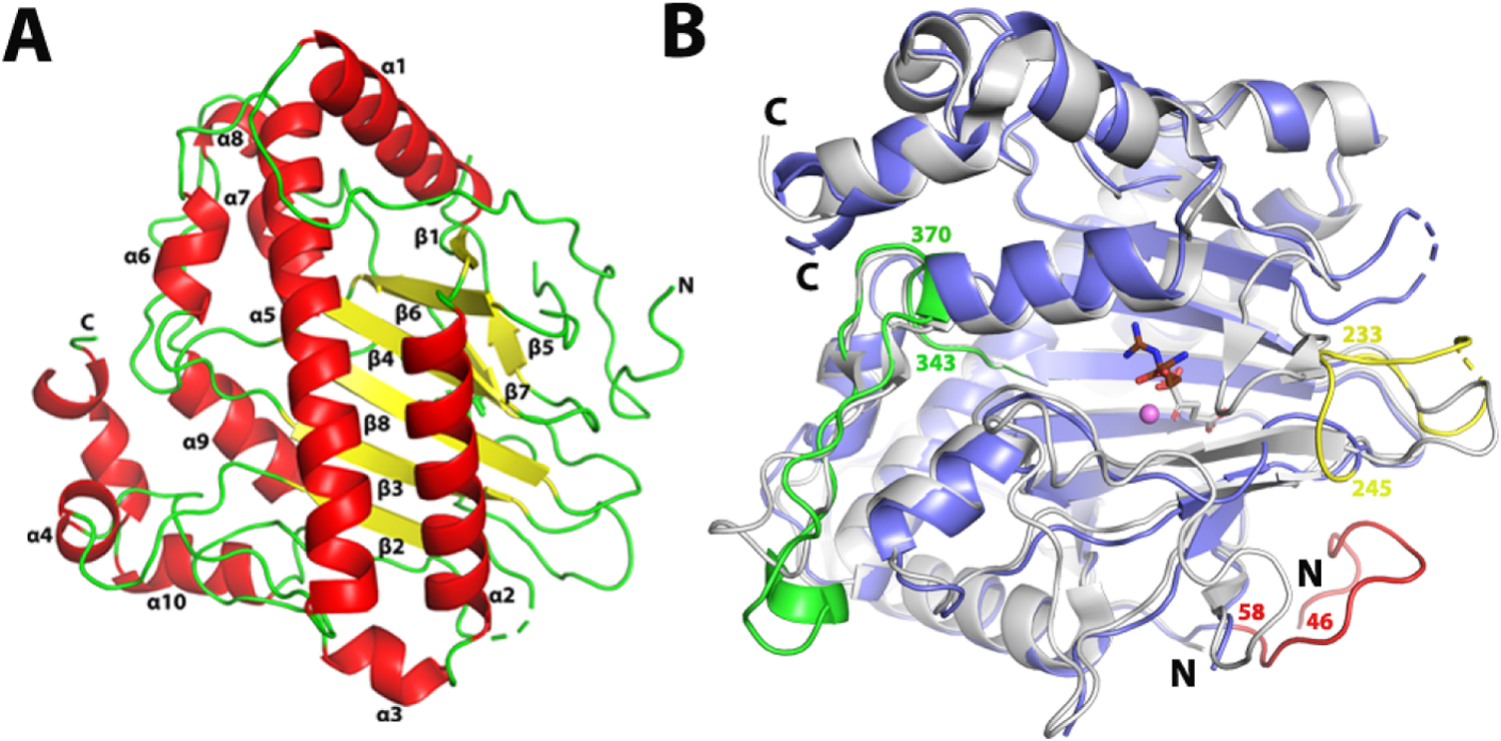
Overall fold of Pd1 EFE and comparison to that of PK2 EFE. (A) Structure of Pd1 EFE·Mn(II)·2OG in cartoon view using chain C that had the least disordered regions. The metal and 2OG are not depicted. β-strands are shown in yellow, α-helices in red, and other regions in green. (B) Overlay of the cartoon views of fungal Pd1 (blue) and bacterial PK2 (gray) versions of EFE. The longer N-terminus and two loops of the Pd1 enzyme are shown in red, yellow, and green colors, respectively. Mn(II), 2OG, and l-Arg are shown for the PK2 EFE (PDB: 5V2Y) as a magenta sphere and gray and brown sticks, respectively. The residue numbers shown in panel B are for the Pd1 enzyme.

The Pd1 EFE structure includes a double-stranded β-helix (DSBH, also known as the jellyroll or cupin fold) core,^52^ an architecture that is widely found in members of the Fe(II)/2OG-dependent oxygenases. We identified eight β-strands, six from the major β-sheet and two from the minor β-sheet, forming the DSBH that is surrounded and stabilized by 10 α-helices (Figure 5A). Among the eight β strands, β1 at the N-terminus extends the major β-sheet at the end of the DSBH, whereas the other N-terminal β strand, β2, extends the other end of the major β-sheet. Among the α helices, α2 and α5 bind across the surface of the major β-sheet and stabilize it. The C-terminal α helices (α8, α9, α10) contribute to the active site for l-Arg binding/interaction. The overall fold of Pd1 EFE is very similar to that of PK2 EFE with a Cα RMSD of 0.6 Å (Figure 5B). The loop between the two β-strands (from 233 to 245, shown in yellow) spanning the 2-His-1-Asp metal binding ligands is longer for the Pd1 EFE. Similarly, the loop between β8 and α8 is longer in Pd1 EFE (shown in green) than for PK2 EFE. Also, we have located the longer N-terminal region of the fungal EFE (shown in red) compared to the shorter PK2 enzyme.

**Figure 6:**
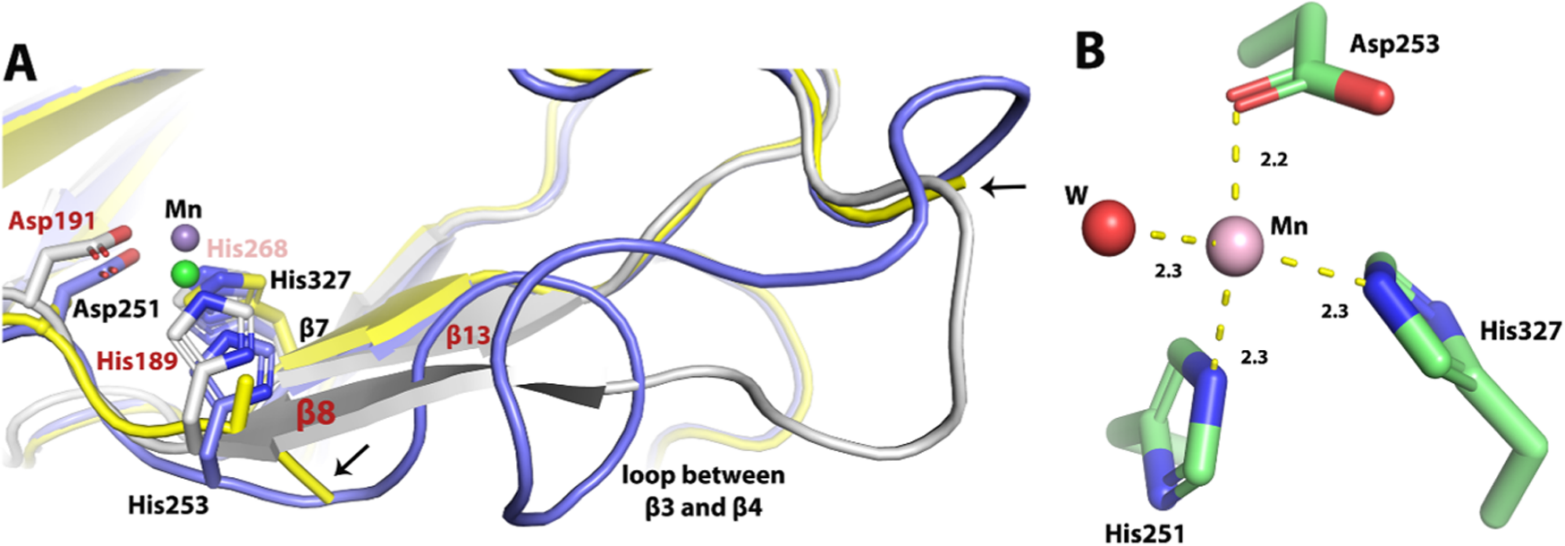
Structural comparison of the metal binding site for PK2 versus Pd1 EFE and closeup view of the Pd1 EFE metal center. (A). Alignment of PK2 EFE·Mn(II)·2OG·l-Arg (gray, PDB ID: 5V2Y, chain A), Pd1 EFE·Mn(II)·2OG (blue, chain C), and the Pd1 EFE apoprotein (yellow, chain A). The disordered region of the Pd1 apoprotein is found between the two black arrows. The bound metals are shown as spheres (green for the Pd1 enzyme and slate for PK2 EFE). 2OG and l-Arg are not shown. Residue numbers and secondary structures are labeled in black and red for the Pd1 and PK2 enzymes, respectively. (B) Metal binding site of the Pd1 EFE·Mn(II)·2OG complex with 2OG not depicted. Distances are shown in Å.

The metal-binding site of Pd1 EFE resembles what is found in most other Fe(II)/2OG-dependent oxygenases, with the metal coordinated by His251 and Asp253, that derive from the loop linking β3 and β4, along with His327 that is situated at the N-terminus of β7. For PK2 EFE, the metal binding residues (His189, Asp191, and His268) derive from β8, the loop connecting β8 to β9, and β13 respectively. Structural alignment of PK2 EFE·Mn(II)·2OG·l-Arg (5V2Y) to the Mn(II)-bound Pd1 EFE and Pd1 EFE apoprotein shows that in place of a β strand containing H189 for PK2 EFE there is a loop that connects β3 and β4 for Pd1 EFE (Figure 6A). This loop and the His251/Asp253 side chains are disordered in the Pd1 EFE apoprotein structure which suggests that the presence of metal is required for the stability of the β3-β4 connecting loop. A single water occupies the position *trans* to His327 in Pd1 EFE·Mn(II)·2OG (Figure 6B).

**Figure 7:**
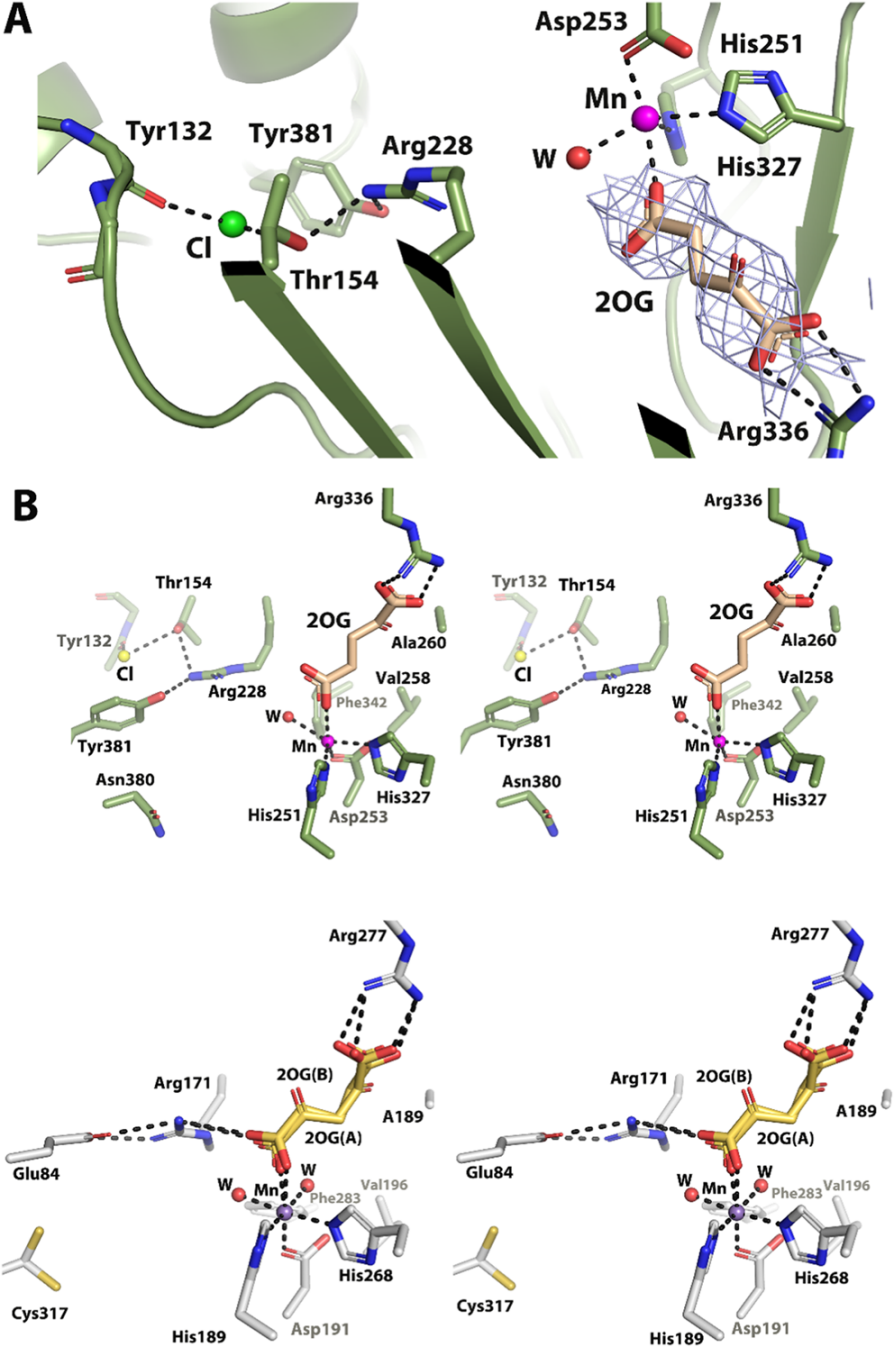
Active site of Pd1 EFE·Mn(II)·2OG and comparison to PK2 EFE·Mn(II)·2OG (PDB: 5V2X). (A) Mn(II), 2OG, and chloride bound to Pd1 EFE. Composite omit map of 2OG (2Fo-Fc, σ = 1) is shown in gray mesh. (B) Comparison of key residues at the active sites of Pd1 EFE·Mn(II)·2OG (green, upper panel) and PK2 EFE·Mn(II)·2OG (gray, lower panel) are shown in stereo view. Magenta and blue spheres represent Mn of Pd1 EFE·Mn(II)·2OG and PK2 EFE·Mn(II)·2OG, respectively. Selected waters are represented as red spheres for the two proteins.

In the Pd1 EFE·Mn(II)·2OG crystal structure, 2OG binds to the metal in a monodentate fashion exclusively with its C5 carboxylate group (Figure 7A). By contrast, either the C1 or the C5 carboxylates of 2OG coordinate the metal in the dual conformations for PK2 EFE·Mn(II)·2OG (Figure 7B). Bound 2OG in the Pd1 protein is stabilized by a salt bridge interaction between Arg336 and the C1 carboxylate group, analogous to the situation in PK2 EFE where Arg277 interacts with the distal carboxyl group. Unlike the situation for PK2 EFE·Mn·2OG (PDB: 5V2X) involving Arg171, the corresponding Arg residue of Pd1 EFE nearer the metallocenter, Arg228, does not interact with 2OG. Rather, in the fungal protein Arg228 interacts with the nearby Tyr381 and Thr154, and an electron density near these residues (insufficiently modeled by water) was assigned as a chloride ion due to the presence of this anion during purification and crystallization (Figure 7A). Important hydrophilic and hydrophobic residues at the active site are similar for PK2 and Pd1 EFEs (Figure 7B),^17^ except that Cys317 in PK2 EFE·Mn(II)·2OG (which exhibits dual conformations) is replaced with a single rotamer of Asn380 in the Pd1 enzyme.

To examine whether the active site Cys317 in PK2 EFE versus Asn380 at the corresponding position in the Pd1 EFE accounted for their significant differences in the protein color after Ni-NTA chromatography, partition ratios, or other properties, we studied the N380C variant of Pd1 EFE that was generated by site-directed mutagenesis of the corresponding gene and purified in the same manner as the nonvariant protein. We showed that the identity of the amino acid at this position was not responsible for the differences between the two enzymes in the color of as-purified proteins and the partition ratio; e.g., the partition ratio of the N380C enzyme was 6.1 ± 1.7 (Figure 4). Like the nonvariant enzyme, the N380C version of Pd1 EFE exhibited an increase with time in ethylene and then plateaued, again likely due to substrate depletion (Figure S7). The kinetic parameters of the variant protein measured for ethylene production while varying l-Arg (*k*_cat_ of 25 ± 3 min^–1^ and *K*_m_ of 140 ± 5 μM) are comparable to those of the wild-type enzyme, so the Cys/Asn residue does not significantly affect the enzyme’s affinity for l-Arg.

For the Pd1 EFE·Mn(II)·2OG structure, residues 137–150 do not move significantly toward the active site when the metal and 2OG are bound. In PK2 EFE this loop acts like a “lid” that covers or shields the active site from the solvent; however, movement of this loop might be required to bind l-Arg. Closing the “lid” for Pd1 EFE would require shifting of a loop connecting β3-β4 (residues 233 to 245) that will necessitate movement of the extra-long N-terminal region. Therefore, it is understandable that binding of l-Arg to Pd1 EFE requires a severe reorientation of the loops, as also predicted by the computational studies described below.

### Computational Studies of Pd1 EFE

Because we were unsuccessful in obtaining the structure of Pd1 EFE·Mn·2OG·l-Arg, either by soaking l-Arg into the Pd1 EFE·Mn·2OG crystals or by cocrystallization, we used computational methods to study this form of the protein. Hence, we modeled the Pd1·Fe(III)·OO^•–^·2OG·l-Arg complex and ran 1 μs MD simulations. The simulations provided a well-equilibrated trajectory with an average root-mean-square deviation (RMSD) of 1.55 Å (Figure S8). The atomistic analysis revealed stable interactions between the C1 carboxylate of 2OG and the guanidinium group of Arg228 and between the C5 carboxylate and guanidium group of Arg336 (Figure S9A) in agreement with the 2OG interactions observed in the PK2 EFE system (Figure S9B).^16,19^ Because the crystal structure of Pd1 EFE·Mn(II)·2OG has the C5 carboxylate of 2OG coordinated to the metal center (Mn), these interactions were not present. The simulations showed interactions between the backbone of Asp253 and the carboxylate of Asp312, consistent with the Pd1 EFE crystal structure. Importantly, the flexibility of the 2OG-bound species is similar for the Pd1 and PK2 systems with congruent RMSD (Figure S10) as a consequence of similar stabilizing interactions.

**Figure 8:**
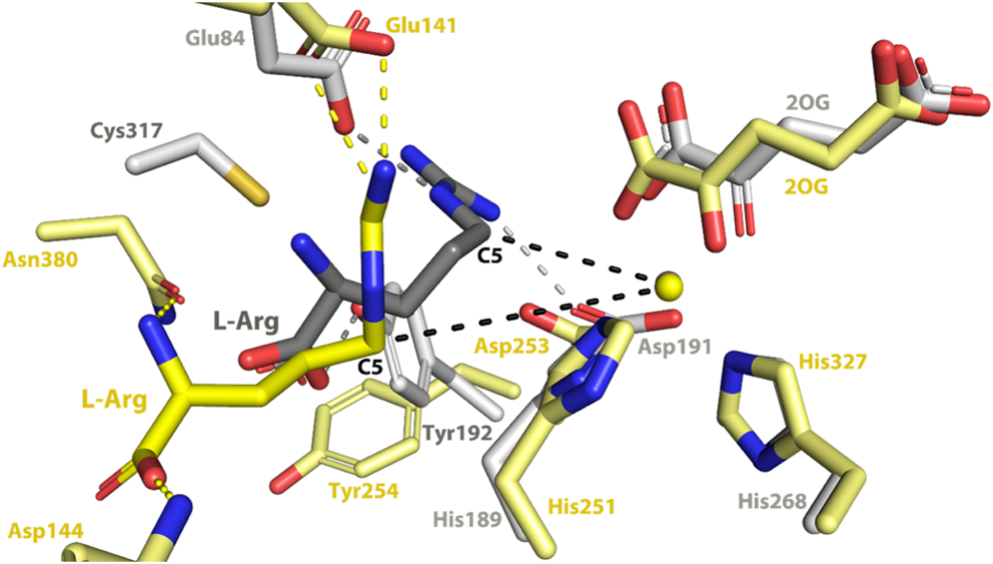
Comparison of the predicted l-Arg binding orientation for Pd1 EFE and the structure of PK2 EFE·Mn(II)·2OG·l-Arg. The computed l-Arg binding site in Pd1 EFE·Fe(II)·2OG·l-Arg (yellow) and the structurally characterized site in PK2 EFE·Mn(II)·2OG·l-Arg (gray) are illustrated. Substrates and residues are shown in stick view and the metals are shown as spheres. The l-Arg is significantly displaced in the fungal enzyme, with a metal to C5 of l-Arg distance of 6.5 Å compared to 4.2 Å in PK2 EFE.

In the Pd1 enzyme, the l-Arg substrate demonstrates greater mobility with higher RMSD than in the PK2 enzyme (Figure S11) and forms fewer stabilizing interactions, averaging around three hydrogen bonds throughout the MD trajectory. In contrast, on average, the PK2 enzyme forms nearly four hydrogen bonds throughout the trajectory, indicating better substrate stabilization. In the PK2 system, the substrate adopts a more constrained L-shaped orientation, whereas in Pd1 EFE, the l-Arg substrate is more relaxed (Figure 8). Although in PK2 EFE, Nε of the l-Arg substrate shows stable hydrogen bonds with Mn(II)-coordinated Asp191, this interaction is predicted to be lost in the Pd1 enzyme (Figure S12A,B). Similarly, the interaction between the side chain hydroxyl group of Thr143 and Nε of l-Arg of Pd1 EFE contrasts with the interaction between the Thr86 and amino group of l-Arg in the PK2 system (Figure S12A,B).^16,19^ The guanidinium group of l-Arg interacts with the Pd1 EFE Glu141 carboxylate, whereas the amino group of l-Arg interacts with the Glu84 carboxylate in the corresponding sequence position of the PK2 EFE (Figure 8).^16,19^ Because of the change in the l-Arg interactions, the substrate binds in a position away from the Fe(III)-center in the Pd1 system compared to the PK2 system (Figure S13). Additionally, we observed two distinct conformations of the l-Arg substrate in the Pd1 system, like what was seen in the PK2 system,^19^ however, with much more frequently represented intermediate states between A and B than in the PK2 enzyme. The Pd1 EFE conformation A (where the C5 of l-Arg faces toward the metal) is significantly more prevalent in the MD trajectory compared to conformation B (where the C5 of l-Arg faces away from the metal), unlike the PK2 system where conformation B is the most populated. However, in the Pd1 system, the C5 of l-Arg is farther from the metal center than in PK2. The average distance between the C5 of l-Arg is 6.4 Å in the Pd1 system, compared to 5.1 Å observed in the PK2 system dynamics (4.2 Å in the crystal structure of PK2 EFE), where conformation B is most populated (Figure S13). Also of interest, Tyr192 was shown to undergo a significant reorientation leading to a twisted peptide bond upon binding of l-Arg to the PK2 enzyme,^16^ whereas the comparable residue Tyr254 of Pd1 EFE does not appear to shift significantly with l-Arg binding.

**Figure 9:**
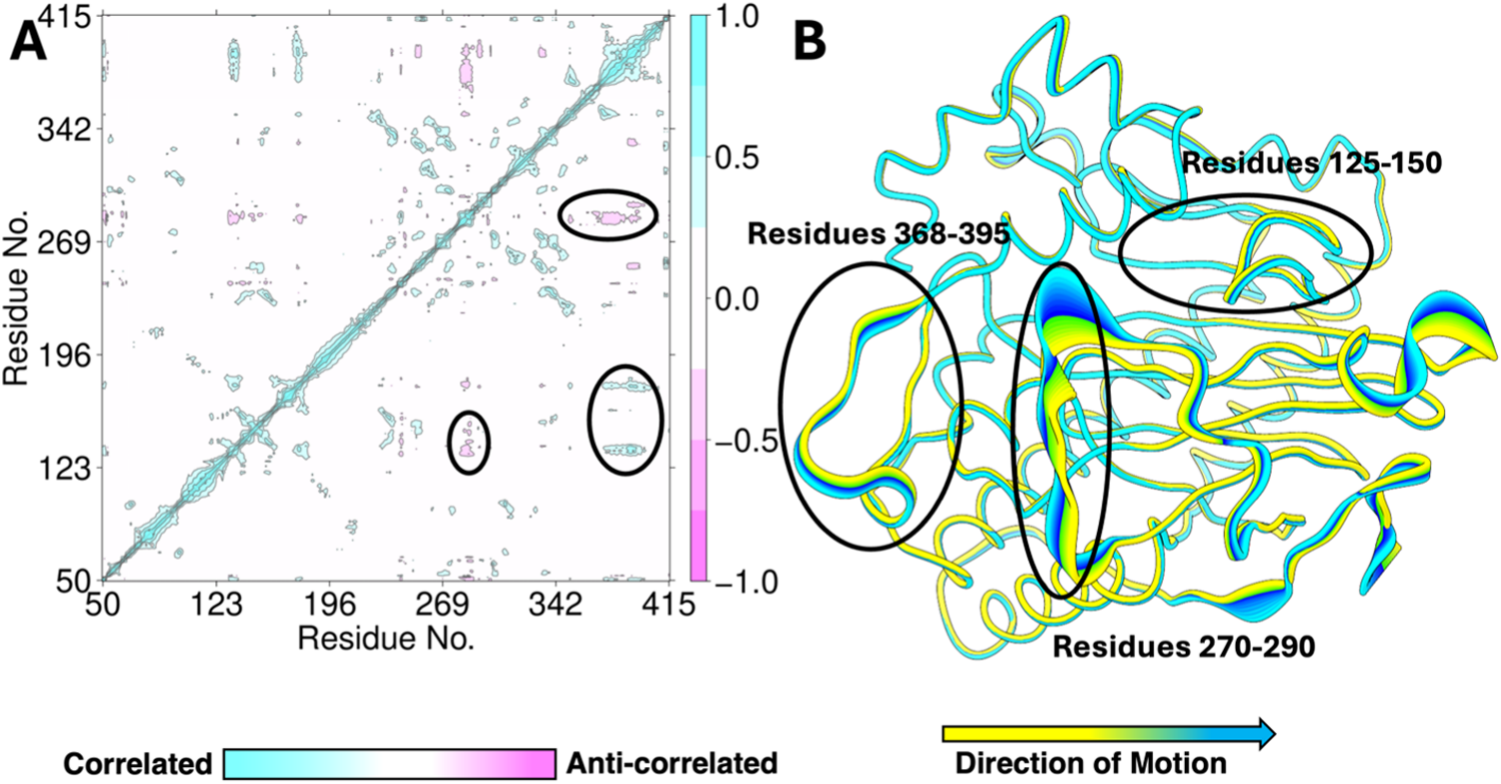
Overall protein dynamics of Pd1 EFE·Fe(III)·OO^•–^·2OG·l-Arg complex. (A) Dynamic cross-correlation matrix shows the regions of correlated and anticorrelated motions in the complex. (B) Principal component analysis indicates the flexible regions of the complex. The circled regions highlight the portions of the enzyme that exhibit important correlated and anticorrelated motions by DCCA. DCCA and PCA of the PK2·Fe(III)·OO^•–^·2OG·l-Arg complex are given in the SI for comparison (Figure S14).

Furthermore, we implemented DCCA analysis to explore the correlated and anticorrelated motions involved in the Pd1 EFE·Fe(III)·OO^•–^·2OG·l-Arg complex. There are correlated/anticorrelated motions in three significant regions of the enzyme, namely, the residues that form (a) the loop between α3 and α4 (125-150), (b) the loop connecting β5 and β6 (270-290), and (c) the region forming α8 and α9 (368-400). Of these three regions, residues 270-290 are disordered in Chain A of the Pd1 EFE crystal structure. Region (b) is anticorrelated with regions (a) and (c), similar to what was observed in the PK2 system (Figures 9A and S14).^19^ Region (c) is correlated with (a) (Figure 9A), in contrast to the anticorrelated motion observed between corresponding regions in the PK2 system.^19^ Importantly, these regions consist of the residues (Glu141, Thr143) that interact with the l-Arg substrate, especially region (a), which is expected to act as a lid for l-Arg binding. Hence, the correlated/anticorrelated motions exhibited by these regions are important for substrate binding and proper orientation. PCA analysis also showed that regions (b) and (c) are highly flexible and seem to be moving away from the active site, resulting in l-Arg displacement in the case of the Pd1 system (Figure 9B). Overall, the MD simulations predicted changes in l-Arg binding due to the changes in its interactions with the Pd1 protein residues and the correlated/anticorrelated motions, which could facilitate a greater level of ethylene production compared to l-Arg hydroxylation in the Pd1 enzyme.

**Figure 10:**
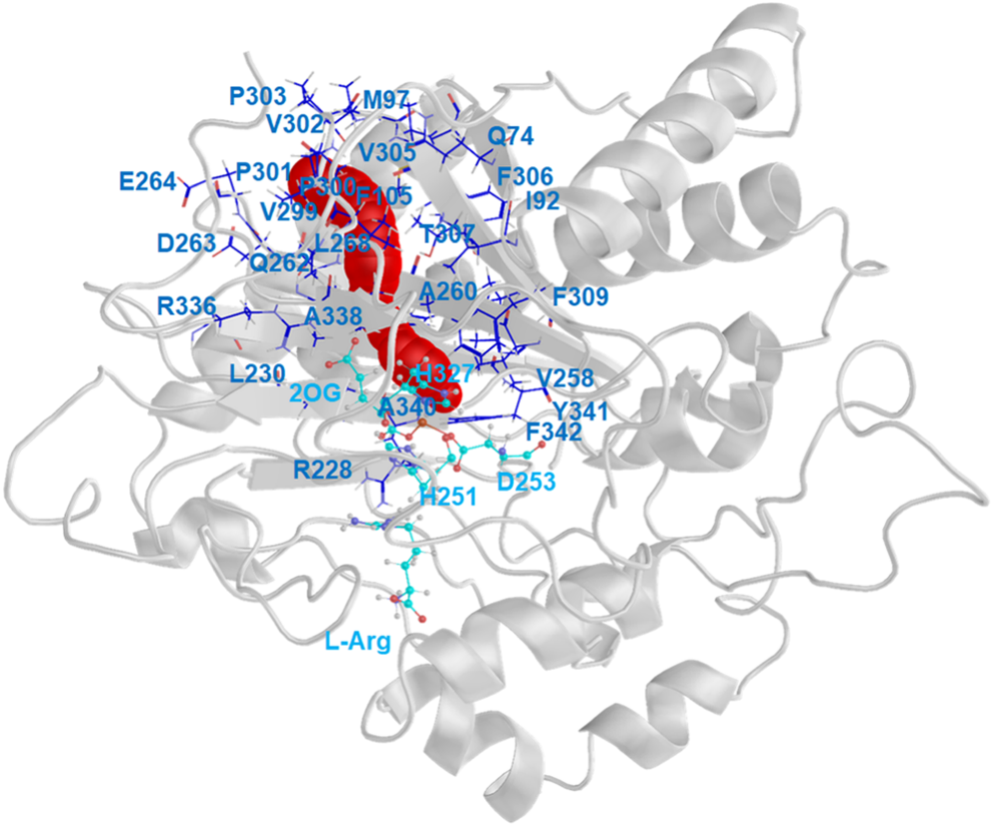
Predicted O_2_ access tunnel to the Pd1 EFE active site. The tunnel is shown in red within the cartoon view of Pd1 EFE, and the residues lining the tunnel are indicated as sticks with blue carbon atoms with labels for the larger side chains. The sticks with cyan carbon atoms are the three side chains that coordinate the metal ion, 2OG, and l-Arg. The identity of the residues is also shown in Figure S17.

To explore the possible O_2_ access tunnels in the Pd1 enzyme, we carried out analysis using CAVER for the structures obtained from the equilibrated portion of the MD. Based on our analysis, we observed a tunnel with a bottleneck radius of 1.11 Å through which the O_2_ can access the Fe center in an offline orientation (Figure 10), similar to the one predicted in the PK2 system, which has a 1.15 Å bottleneck radius.^22^ The tunnel residues that act as gatekeepers in Pd1 EFE include Asp263, Glu264, Pro303, and Phe105 (Figure S15), while in PK2 EFE, residues Gln200, Asp202, Thr243, and Val246 form the gatekeepers for the tunnel. At the active site of Pd1 EFE, the tunnel is surrounded by hydrophobic residues, including Arg228, Val258, Phe309, Ala340, and Phe342 (Figure S16), while in PK2, the active site tunnel residues include Val196, Phe250, Ala281, and Phe283.^22^ The residues that form the tunnel in the Pd1 and PK2 systems are provided in the SI (Figure S17).

## Conclusions

This work extends our understanding of the biochemical properties and is the first to describe the structure of a eukaryotic version of the EFE. Although clearly related in sequence and structure to the well-characterized bacterial PK2 enzyme, Pd1 EFE has several distinct features. One suggested distinction at the l-Arg binding site is an Asn residue in Pd1 EFE compared to a Cys residue at the active site of PK2 EFE. We created and used the N380C variant to show that, while the variant Pd1 enzyme activity is decreased compared to the nonvariant enzyme, the substitution does not account for the following distinct properties of these EFE proteins.

**Figure 11:**
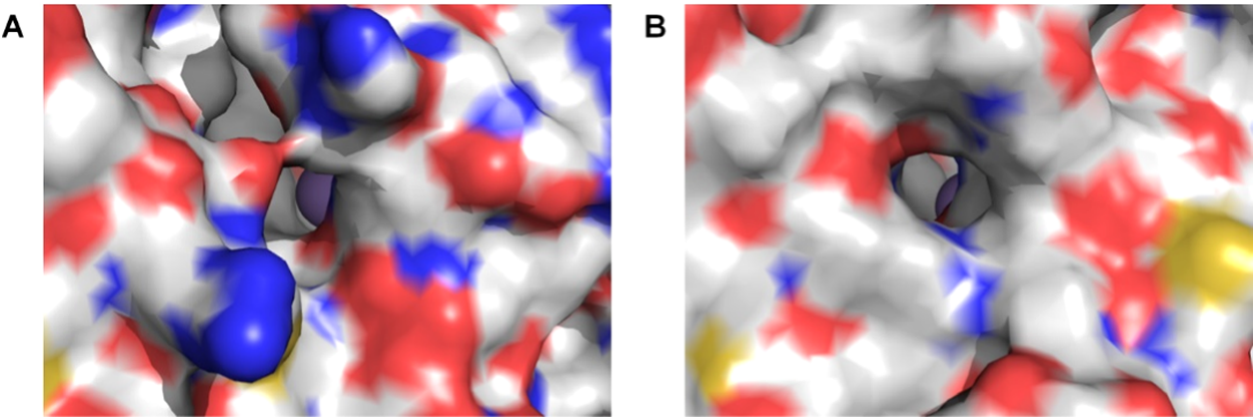
Active site access views of Pd1 and PK2 EFE proteins. Surface views of (A) Pd1 EFE and (B) PK2 EFE, with yellow showing sulfur atoms, red for carboxylates, and blue for positively charged residues. Mn(II) is a gray sphere in each panel.

As purified using a Ni-NTA column, PK2 EFE is blue in color and possesses near stoichiometric amounts of nickel that is most likely acquired from the resin, whereas Pd1 EFE has a low metal ion content and is faint yellow in color that might correspond to low levels of bound ferric ion. The crystal structures of these enzymes reveal a more open access site for the Pd1 enzyme that could account for ease of nickel dissociation as well as a nearby positive surface charge that could reduce nickel cation binding to the enzyme active site (Figure 11).

The anaerobic UV–visible difference spectrum of Pd1 EFE suggests that 2OG chelates the active site Fe(II) in the absence of l-Arg to produce three low energy MLCT transitions, with little change occurring when l-Arg is added. These results contrast with the situation for PK2 EFE where the difference spectrum for enzyme with 2OG is very weak but is several-fold enhanced by the addition of l-Arg to yield less-well-distinguished MLCT transitions at greater energy. Given their similarities in active sites, the basis for the shift in wavelengths is unclear but is presumed to reflect small changes in the dihedral angle between the carbonyl and C1 carboxyl group of 2OG. The increase in intensity induced by l-Arg for PK2 EFE was attributed to a transition from primarily monodentate coordination to chelation of the metal accompanied by a significant shift of Tyr192.^16^ Such a shift of the analogous residue in Pd1 EFE, Tyr254, is not predicted to occur and may lead to chelation of Fe(II) in Pd1 EFE whether l-Arg is present or not. Surprisingly, however, the crystal structure of Pd1 EFE·Mn(II)·2OG depicts primarily monodentate coordination of 2OG in a reversed conformation, i.e., with the C5 carboxylate rather than C1 carboxylate bound to Mn(II). We hypothesize that the crystal lattice prevents 2OG from binding as a chelate species and this may also prevent l-Arg binding.

We showed that Pd1 EFE simultaneously generates ethylene, succinate, and P5C, like the PK2 enzyme. Moreover, we confirmed the expectation that both PK2 and Pd1 EFEs catalyze the uncoupled decomposition of 2OG to produce succinate and CO_2_ when l-Arg is absent, and we demonstrated that the fungal enzyme, like PK2 EFE, forms small amounts of 3-HP. Although an ethylene/succinate ratio of 2 was originally reported for PK2 EFE,^10^ we found almost a 2-fold larger partition ratio for this enzyme based on the production levels for ethylene and P5C. Notably, we observed an even greater partition ratio for the Pd1 EFE. Thus, the fungal enzyme is better suited for ethylene production because it would result in less nondesirable decomposition of l-Arg by cells. The greater partition ratio observed for Pd1 EFE over PK2 EFE may relate to the greater distance predicted between Fe(II) and the C5 of l-Arg for the former enzyme, as predicted by our computational analyses. A resulting slower rate of l-Arg hydroxylation could lead to a corresponding increase in reactivity leading to ethylene production.

Two important structural and conformational considerations might be relevant to the different ethylene:P5C ratios for Pd1 and PK2 versions of EFE: (i) As described above, the MD simulation shows that there are changes in the binding interactions and the flexibility of the l-Arg substrate in the Pd1 system with respect to PK2 EFE, however, the interactions of the 2OG in Pd1 are consistent in both systems. The diverse binding interactions and flexibilities of l-Arg in Pd1 and PK2 versions of EFE might influence differentially the hydroxylation of l-Arg in both enzymes—specifically the higher hydroxylation activity in PK2 EFE and the lower one in the Pd1 enzyme. (ii) On the other hand, the consistent maintenance of the 2OG interactions in both systems might be responsible for the sustained ethylene production in both enzymes. The combined effect of both factors could explain the greater ethylene:P5C ratio in Pd1 EFE compared to the PK2 enzyme. However, further experiment-correlated Quantum Mechanics/Molecular Mechanics reaction mechanisms studies of Pd1 would be necessary to further test this hypothesis. Regardless of the factors controlling the partition ratio, one important observation from this work is that the Pd1 enzyme exhibits a greater value than the PK2 EFE; thus, heterologous production of EFE for the purpose of ethylene production would benefit by making use of the Pd1 enzyme due to its decreased metabolism of the essential cellular metabolite l-Arg.

In conclusion, we have made significant advances in the characterization of a fungal and first eukaryotic version of EFE. We confirmed that it catalyzes the four reactions shown in Figure 1. This work sets the stage for additional investigations to optimize the partition ratio of the enzyme to produce ethylene for industrial purposes.

## Supporting Information Available

EFE sequence alignments, strain Pd1 enzyme purity and size, color differences of PK2 versus Pd1 EFEs, 3-HP production, ethylene and P5C kinetics plots, kinetics of the N380C variant, an electron density map illustrating a portion of the Pd1 EFE backbone and its residues and Mn(II) omit maps, MD dynamics and geometries, and analysis of the O_2_ access tunnel (Figures S1–S17); optimized MS/MS settings, analyte retention times and *m*/*z*, crystallization statistics, metal contents, product levels for varied conditions, and RMSD of the four chains (Tables S1–S6) (PDF)
