# Supplementary material for: Biochemical, Structural, and Conformational Characterization of a Fungal Ethylene-Forming Enzyme
Source: Biochemistry. 2025 Mar 7;64(9):2054–67. doi: 10.1021/acs.biochem.5c00038 (PMC12060275; doi:10.1021/acs.biochem.5c00038)
Supplement: Supplementary file 1 — bi5c00038_si_001.pdf [file bi5c00038_si_001.pdf]

Supporting Information for

Biochemical, Structural, and Conformational Characterization of a Fungal Ethylene-Forming Enzyme

Shramana Chatterjee,[1] Joel A. Rankin,[1,5] Mark A. Farrugia,[1] Simahudeen Bathir J. S. Rifayee,[2] Christo Z. Christov,[2,*] Jian Hu,[3,4,*] and Robert P. Hausinger[1,3*]

[1]Department of Microbiology, Genetics, and Immunology, Michigan State University, East Lansing, Michigan 48824, United States

[2]Department of Chemistry, Michigan Technological University, Houghton, Michigan 49931, United States

[3]Department of Biochemistry and Molecular Biology, Michigan State University, East Lansing, Michigan 48824, United States

[4]Department of Chemistry, Michigan State University, East Lansing, Michigan 48824, United States

[5]Present Address: Department of Biochemistry Molecular Biology and Biophysics, University of Minnesota, Minneapolis, Minnesota 55108, United States

[*]To whom correspondence should be addressed: christov@mtu.edu, hujian1@msu.edu, hausinge@msu.edu

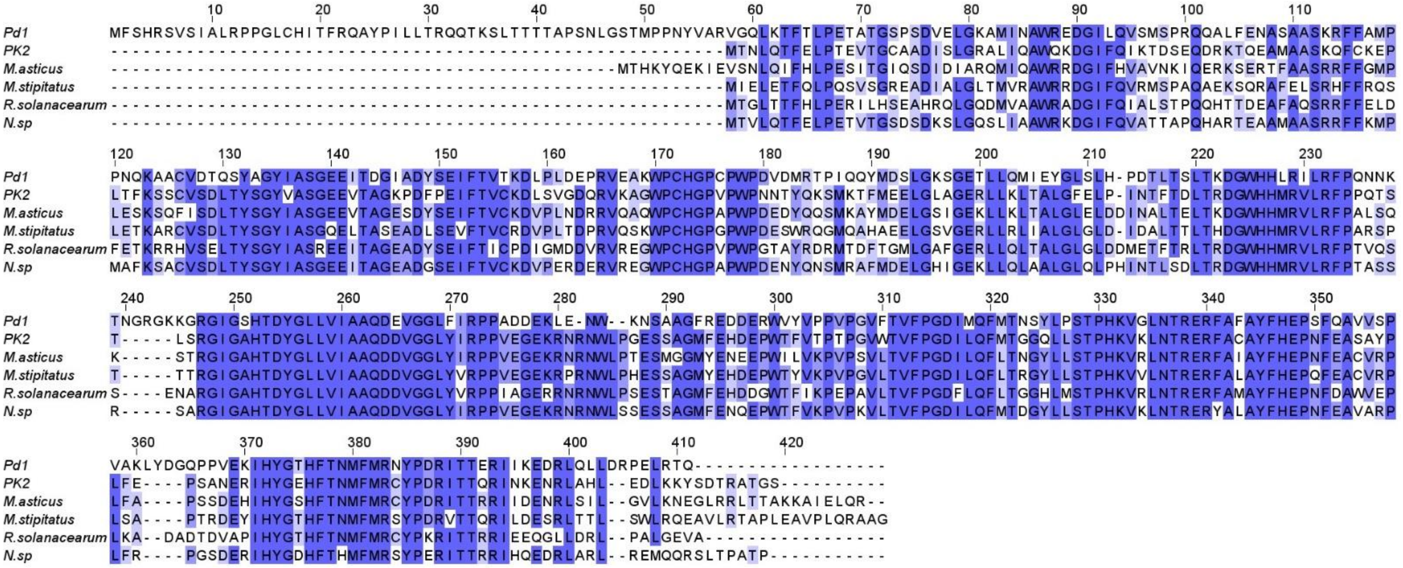

Figure S1. Sequence alignment selected EFEs. Sources: *Penicillium digitatum* strain Pd1 (*Pd1*), *Pseudomonas savastonoi* strain PK2 (*PK2*), *Microcoleus asticus* (*M.asticus*)*, Myxococcus stipitatus* DSM 14675 (*M.stipitatus*)*, Ralstonia solanacearum* (*R.solanacearum*), *Nostoc* sp. ATCC 43529 (*N.sp*). Residues are shaded in blue according to percent sequence identity. The sequence alignment was produced using T-Coffee (http://tcoffee.crg.cat/apps/tcoffee/do:regular).

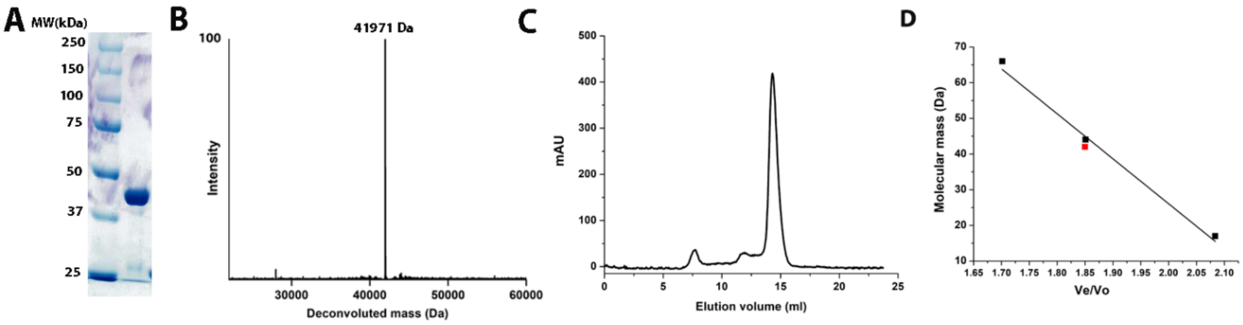

Figure S2. Purity and size of *P. digitatum* strain Pd1 EFE. (A) The homogeneity and an estimate of the subunit size for the thiol-treated and denatured Pd1 EFE was determined by SDS-PAGE (12% acrylamide gel) after staining with Coomassie blue. The molecular weights (MW) indicated at the left side of the gel were obtained using Precision Plus Protein All Blue Prestained Protein markers from Bio-Rad. (B) The precise subunit size of Pd1 EFE was determined by ESI-MS. (C) The native size of the Pd1 EFE apoprotein was determined by SEC (Superdex 200 Increase 10/300 GL) as 42 ± 7 kDa. The absorbance at 280 nm was plotted vs. elution volume and indicated primarily a monomeric form in solution. (D) Plot of the molecular mass in kDa of albumin (66 kDa), ovalbumin (45 kDa), and myoglobin (17 kDa) standards (black dots) vs. the ratio of their elution volume/void volume (V_e/V_O) compared to Pd1 EFE (red dot).

A

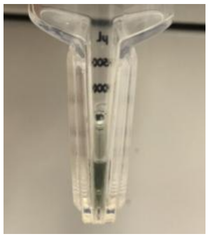

B

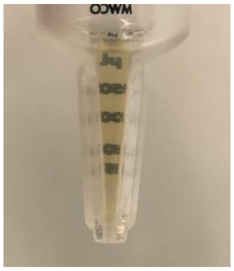

C

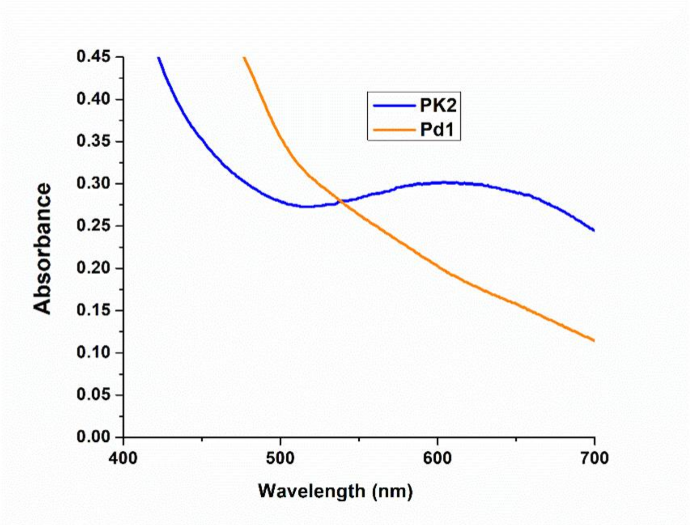

Figure S3. Chromophores associated with the PK2 and Pd1 EFEs after purification using Ni-NTA chromatography. **(**A) Blue color of PK2 EFE. (B) Yellow color of Pd1 EFE. (C) Visible spectra of as-purified Pd1 and PK2 EFEs. Proteins were present in 50 mM NaH_2PO_4, pH 8.0, 500 mM NaCl, 10 mM imidazole. The spectra were obtained using 25 mg mL^{-1} of PK2 and 22 mg mL^{-1} of Pd1, then normalized to the same concentration.

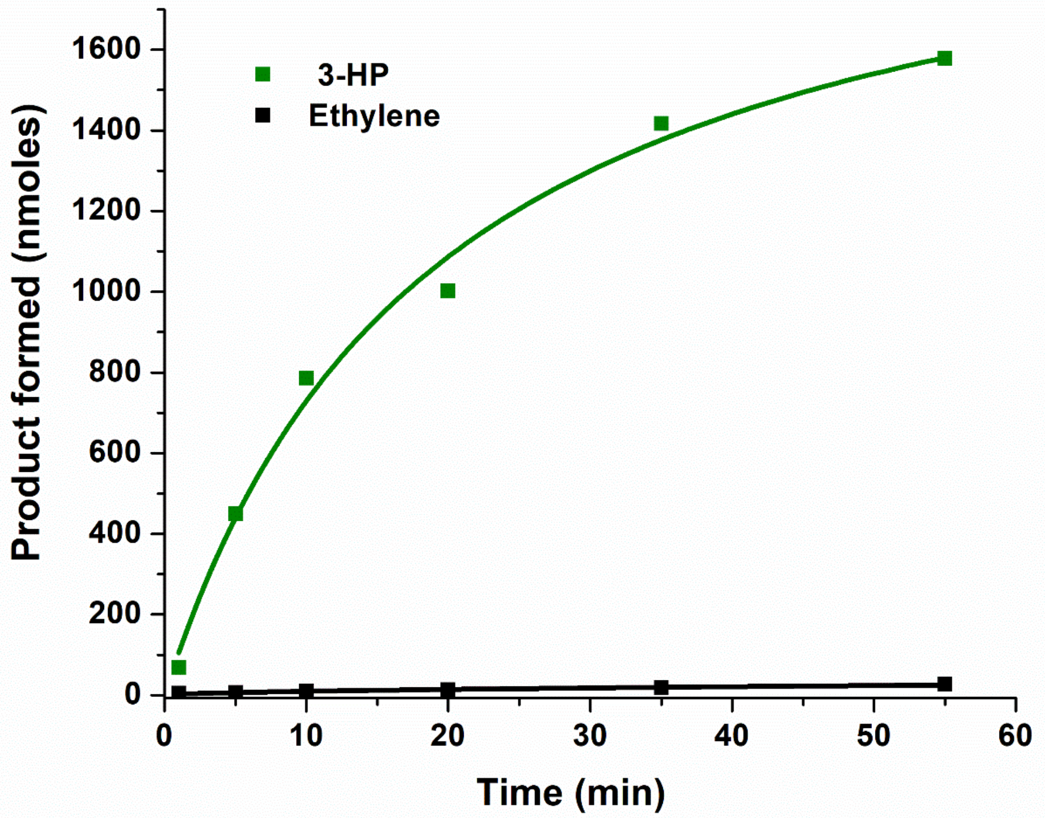

Figure S4. Time-dependent production of 3-HP and ethylene by the A198V variant of PK2 EFE. The formation of ethylene (black) and 3-HP (green) were monitored for the A198V variant of PK2 EFE (60 μM) in a 0.3 mL assay mixture containing 25 mM HEPES buffer (pH 7.5) with 6.67 mM 2OG, 6.67 mM L-Arg, 0.4 mM $Fe(NH_4)_2(SO_4)_2$, and 0.8 mM L-ascorbic acid, then terminated at the indicated time points by adding 0.9 mL of acetonitrile. Production of ethylene and 3-HP were monitored by GC and MS after derivatization with 4-BNMA, respectively. Nonlinear fitting was performed in Origin 8 software.

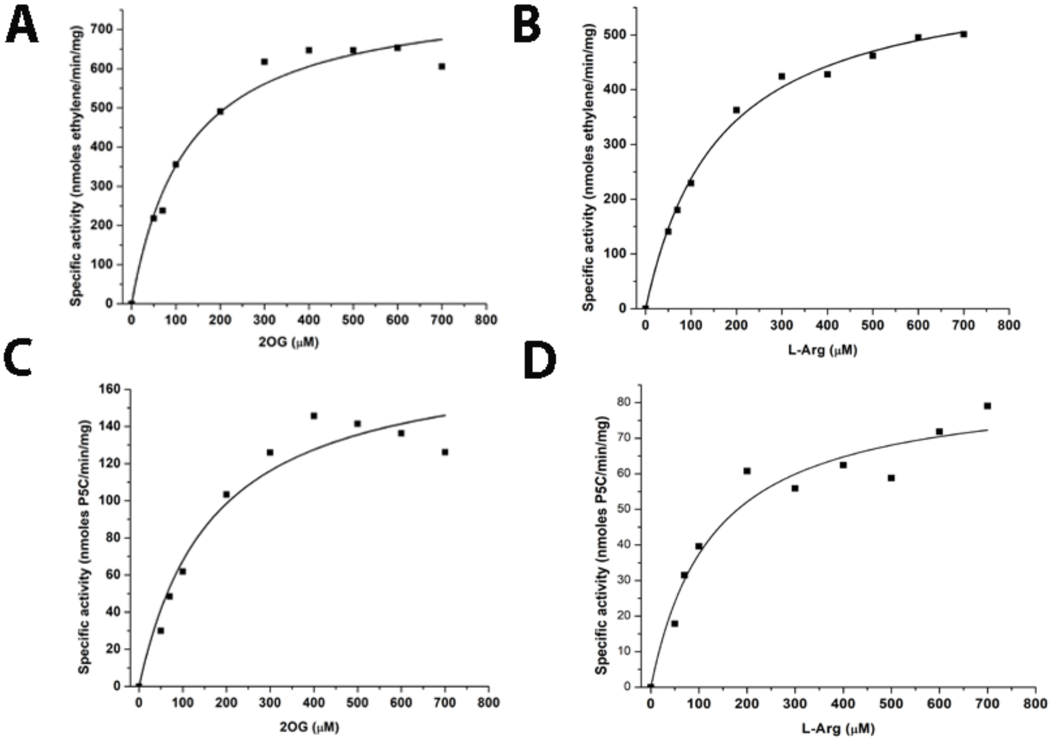

Figure S5. Pd1 EFE kinetics analysis. (A) Ethylene generation as a function of 2OG concentration while maintaining 500 μM L-Arg or (B) as a function L-Arg concentration while maintaining 500 μM 2OG. (C) P5C production as a function of 2OG concentration with 500 μM L-Arg or (D) as a function of L-Arg concentration using 500 μM 2OG. In each case, EFE was added to a final concentration of 125-200 nM in 25 mM HEPES buffer (pH 7.5) containing 0.2 mM $Fe(NH_4)_2(SO_4)_2$, 0.4 mM L-ascorbic acid, and the indicated concentrations of 2OG and L-Arg, then assayed at room temperature (22 ± 1 °C). Non-linear fitting was performed in Origin 8 software.

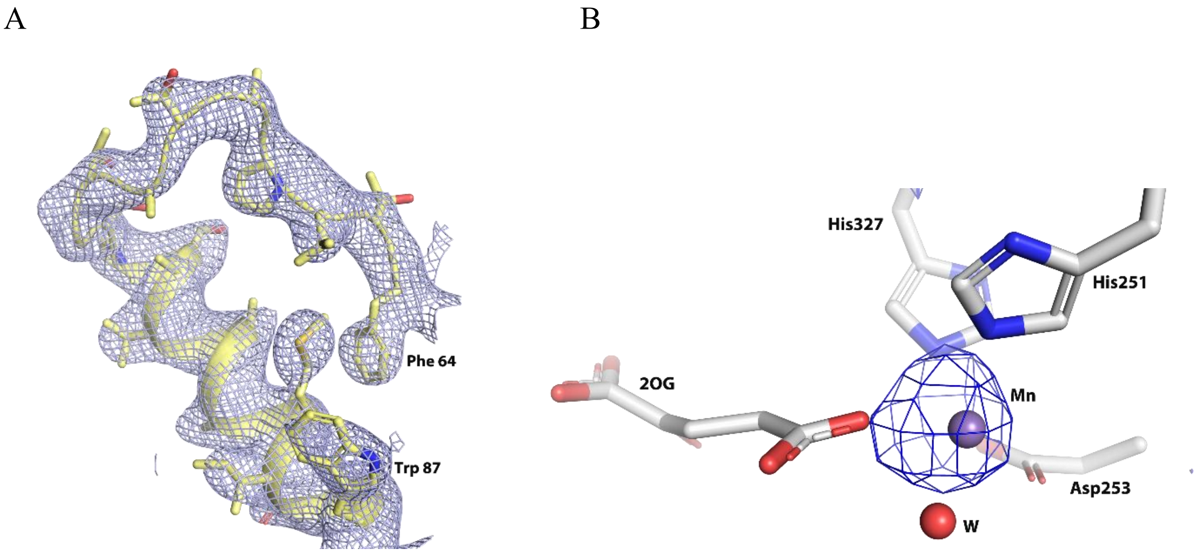

Figure S6. (A) Electron density map (2Fo-Fc, σ = 1) of residues 64 to 87 of chain A. (B) Omit map (σ = 5) for Mn in chain C.

A

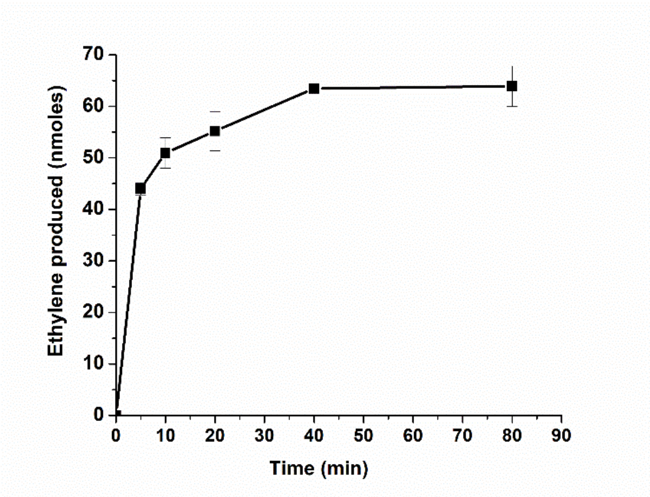

B

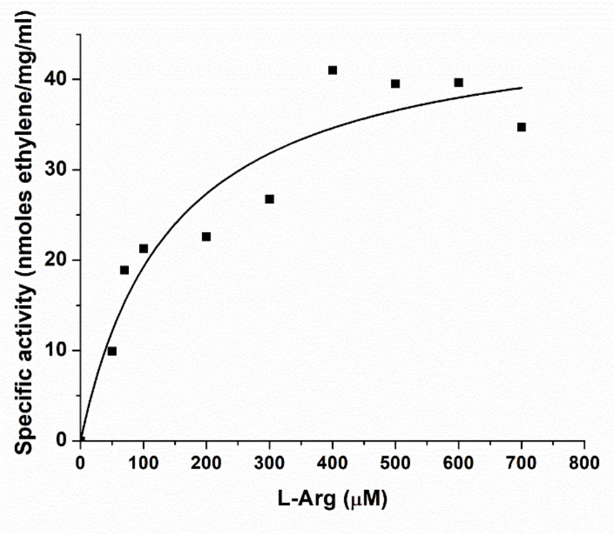

Figure S7. Time dependency and L-Arg concentration dependence of the kinetics for the N380C variant of Pd1 EFE. (A) Ethylene production as a function of time. Freshly purified N380C Pd1 EFE (0.0127 mg/mL, final concentration) was incubated with 0.5 mM 2OG, 0.5 mM L-Arg, 0.2 mM $Fe(NH_4)_2(SO_4)_2$, and 0.4 mM L-ascorbic acid in 2 mL of 25 mM HEPES buffer (pH 7.5) at room temperature (22 ± 1 ^{o}C) and quenched with 0.1 M HCl at the indicated timepoints. (B) Effect of L-Arg concentration on the ethylene forming reaction, with the other conditions as in panel A. Reactions were quenched after 40 min. The data represent three technical replicates of a single biological sample.

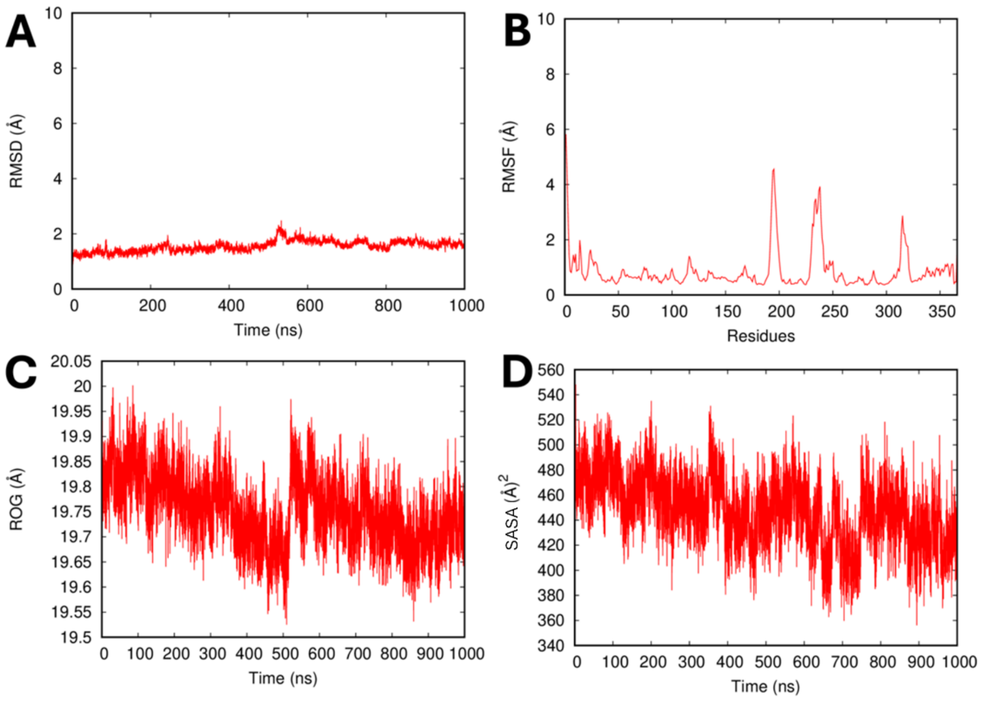

Figure S8. Molecular dynamics analysis of the Pd1 EFE·Fe(III)·$OO^{\bullet -}$·2OG·L-Arg complex. (A) The root mean square deviation (RMSD) of the dynamics suggests the system is equilibrated, (B) the root mean square fluctuation (RMSF) of the system identifies flexible regions, (C) Radius of gyration (ROG) shows the stability of the overall protein fold, and (D) Solvent accessible surface area (SASA) implies that the system is equilibrated.

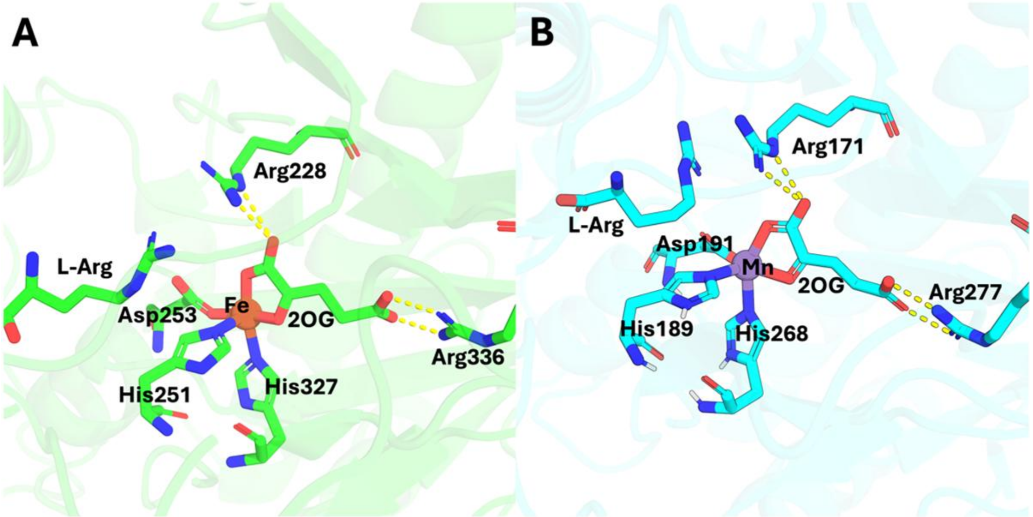

Figure S9. Hydrogen bonds that stabilize 2OG in the (A) Pd1 EFE·Fe(III)·OO$^{\bullet-}$·2OG·L-Arg MD simulations and (B) PK2 EFE·Mn(II)·2OG·L-Arg crystal structure.

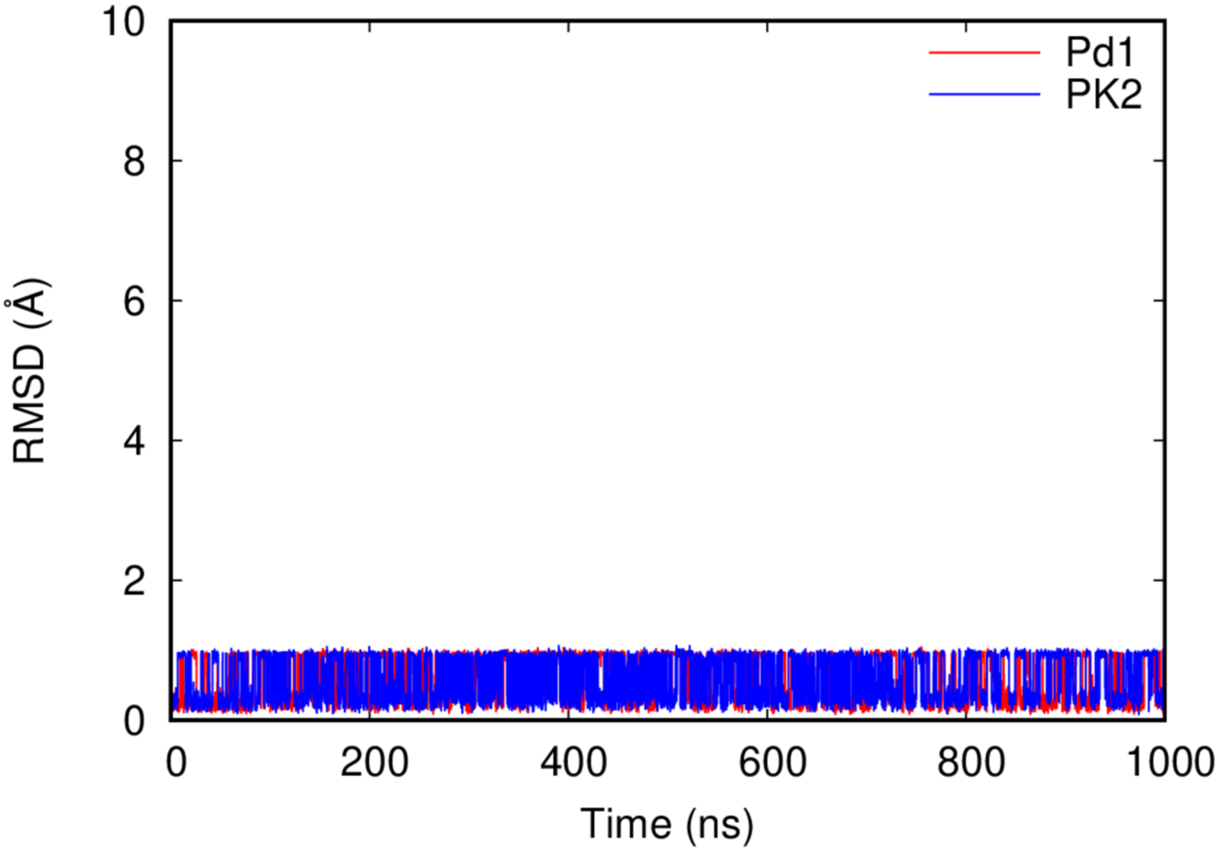

Figure S10. RMSD of the 2OG substrate in the Pd1 EFE·Fe(III)·OO$^{\bullet-}$·2OG·L-Arg and the PK2 EFE·Fe(III)·OO$^{\bullet-}$·2OG·L-Arg complex MD simulations show similar flexibility.[1]

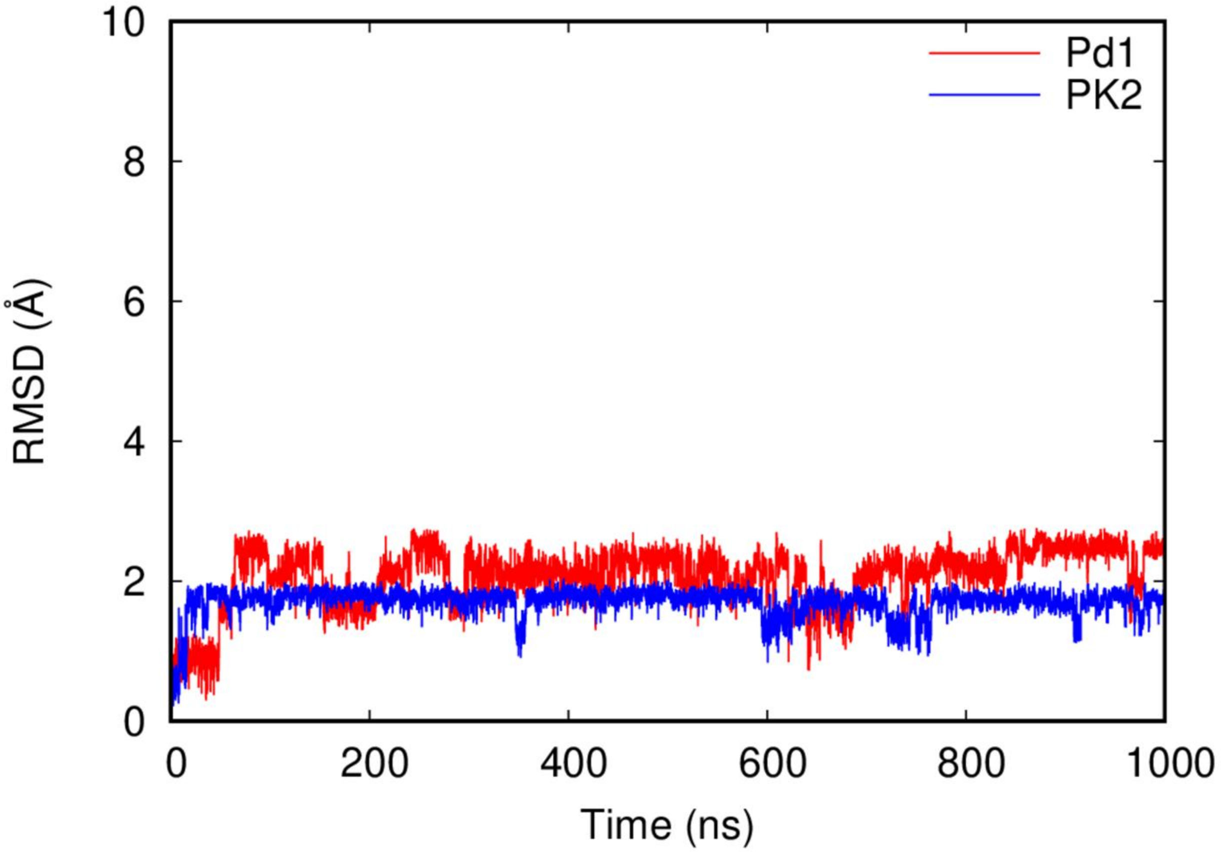

Figure S11. RMSD of L-Arg substrate in the Pd1 EFE·Fe(III)·$OO^{\bullet -}$·2OG·L-Arg and the PK2 EFE·Fe(III)·$OO^{\bullet -}$·2OG·L-Arg complex.[1] The MD simulations show more mobility of L-Arg in the Pd1 enzyme.

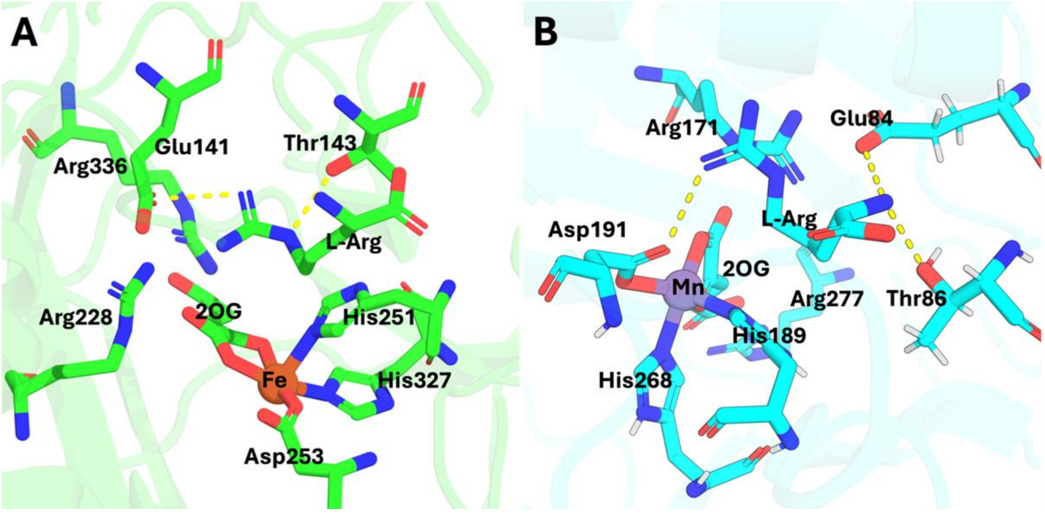

Figure S12. Interactions that stabilize L-Arg in (A) Pd1 EFE MD simulations and (B) PK2 EFE crystal structure.

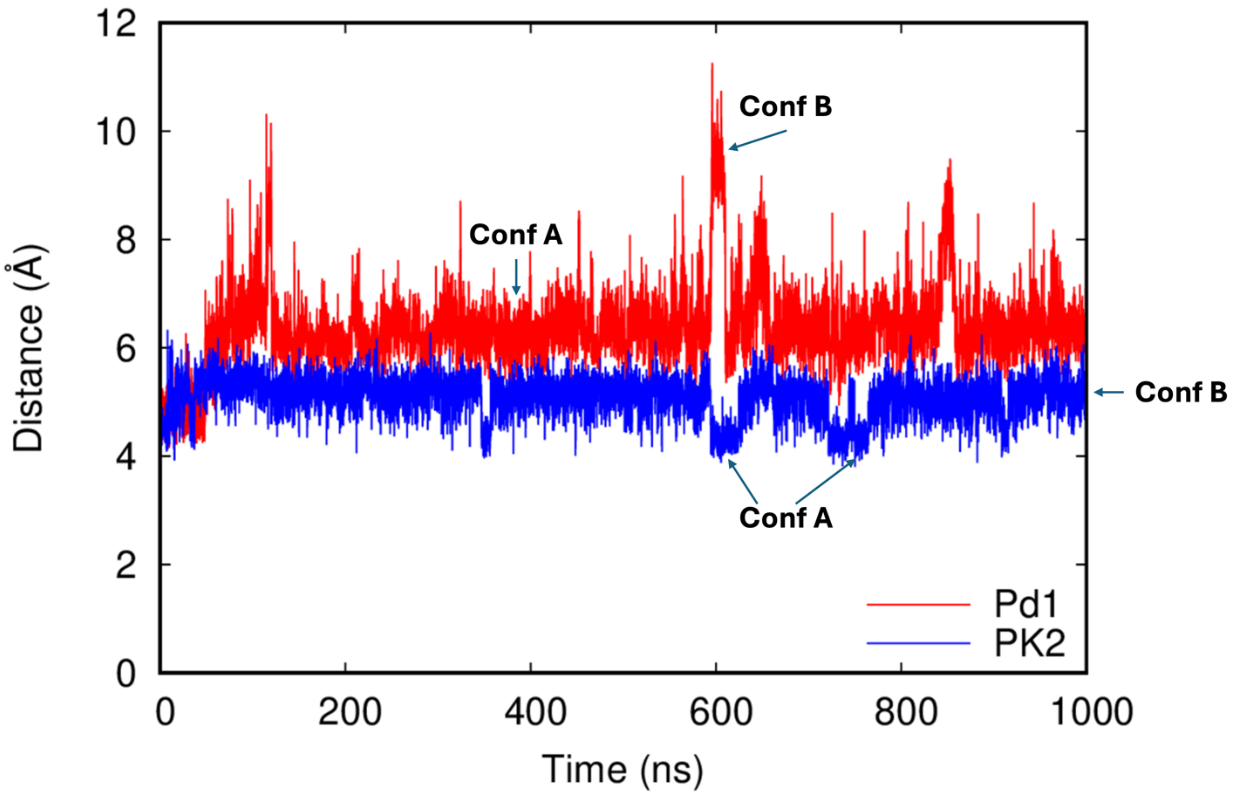

Figure S13. Plot depicting the distance between Fe and C5 of L-Arg during MD simulations shows the changes in L-Arg binding in the Pd1 system (red) compared to PK2 EFE (blue).

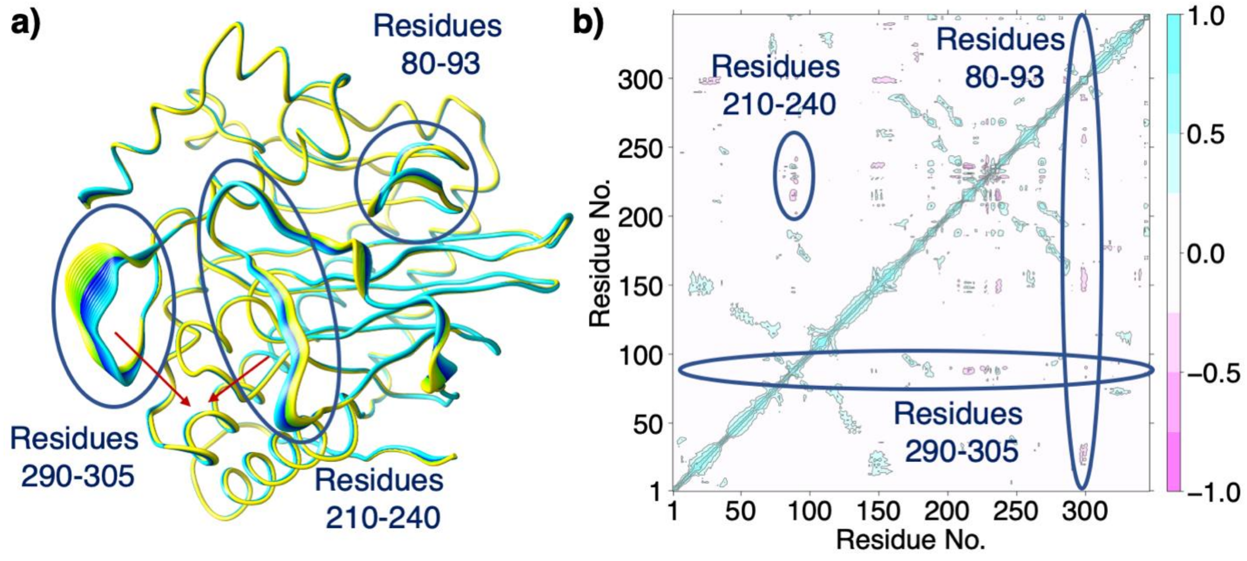

Figure S14. (a) PCA plot showing the flexible regions of the enzyme in PK2 EFE·Fe(III)·OO$^{\bullet-}$ ·2OG·L-Arg complex dynamics. (b) DCCA plot showing the correlated and anticorrelated region of the PK2 EFE·Fe(III)·OO$^{\bullet-}$·2OG·L-Arg complex. Reproduced from Chaturvedi et al.[1]

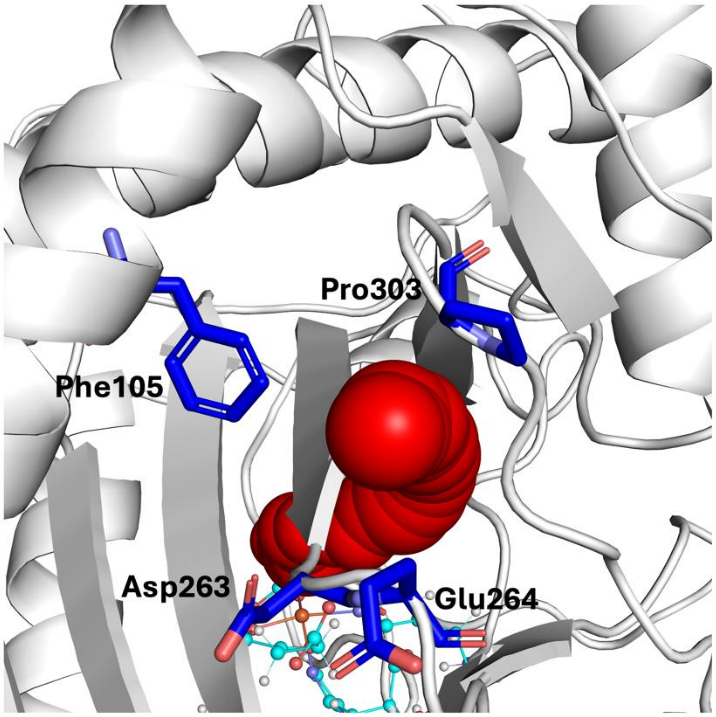

Figure S15. Entry gate residues for the O_2 tunnel in Pd1 EFE.

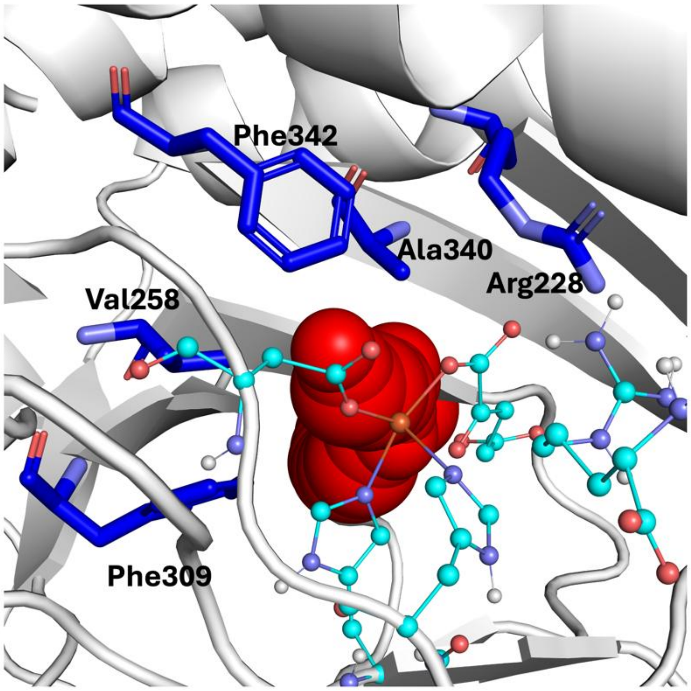

Figure S16. Active site residues surrounding the O_2 tunnel in Pd1 EFE.

```
PK2     ---------------------------------------------------------MTNLQTFELPTEVTGCAADISLG   23
Pd1     MFSHRSVSIALRPPGLCHITFRQAYPILLTRQQTKSLTTTTAPSNLGSTMPPNYVARVGQLKTFTLPETATGSPSDVELG   80

PK2    RALIQAWQKDGIFQIKTDSEQDRKTQEAMAASKQFCKEPLTFKSSCVSDLTYSGYVASGEEVTAGKPDFPEIFTVCKDLS  103
Pd1    KAMINAWREDGILQVSMSPRQQALFENASAASKRFFAMPPNQKAACVDTQSYAGYIASGEEITDGIADYSEIFTVTKDLP  160

PK2   VGDQRVKAGWPCHGPVPWPNNTYQKSMKTFMEELGLAGERLLKLTALGFELPINTFTDLTRDGWHHMRVLRFPPQTST--  181
Pd1   LDEPRVEAKWPCHGPCPWPDVDMRTPIQQYMDSLGKSGETLLQMIEYGLSLHPDTLTSLTKDGWHHLRILRFPQNNKTNG  240

PK2   ---LSRGIGAHTDYGLLVIAAQDDVGGLYIRPPVEGEKRNRNWLPGESSAGMFEHDEPWTFVTPTPGVWTVFPGDILQFM  258
Pd1   RGKKGRGIGSHTDYGLLVIAAQDEVGGLFIRPPADDEKLE-NWK--NSAAGFREDDERWVYVPPVPGVFTVFPGDIMQFM  317

PK2   TGGQLLSTPHKVKLNTRERFACAYFHEPNFEASAYPLF-----EPSANERIHYGEHFTNMFMRCYPDRITTQRINKENRL  333
Pd1   TNSYLPSTPHKVGLNTRERFAFAYFHEPSFQAVVSPVAKLYDGQPPV-EKIHYGTHFTNMFMRNYPDRITTERIIKEDRL  396

PK2   AHLE--DLKKYSDTRATGS  350
Pd1   QLLDRPELRTQ--------  407
```

Figure S17. Residues lining the O_2 tunnels in PK2 and Pd1 EFEs. The organellar targeting region highlighted in yellow was deleted from the construct for Pd1 EFE, so that after TEV cleavage the protein initiated with Ser from the cleavage site, a His-Met linker, then continued at Leu37. The green highlights compare the residues that line the O_2 tunnel 1 in PK2 EFE versus those lining the O_2 tunnel in Pd1 EFE.

Table S1. Optimized MS/MS settings in negative ion electrospray mode.

Analyte	Parent mass (Da)	Daughter mass (Da)	Cone voltage (V)	Collision voltage (V)
Succinic acid	117.0	73.0	-26	-11
2-oxoglutaric acid	145.0	101.0	-18	-11

Table S2. Analyte retention times and *m/z*.

Analyte	Retention time (min)	*m/z*	Scheme
Succinic acid	6.2	483.01	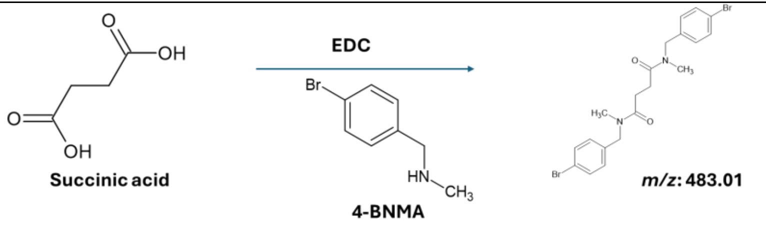
2-oxoglutaric acid	6.5	511.01	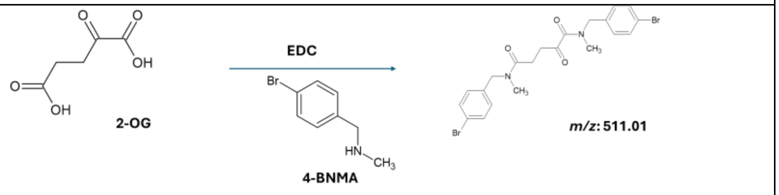
3-HP	4.5	272.02	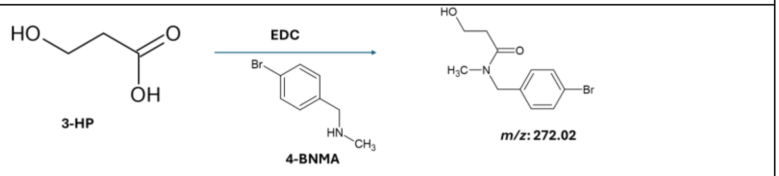

Table S3. Crystallization statistics for Pd1 EFE apoprotein and the Pd1 EFE·Mn(II)·2OG complex

**Pd1 EFE crystals**	**Apoprotein**	**Mn(II)·2OG bound**
**Data collection**		
Beamline	LS-CAT 21-ID-D	LS-CAT 21-ID-D
Wavelength (Å)	1.127	1.127
Space group	P 21 21 21	P 21 21 21
Unit cell a, b, c (Å)<br>α, β, γ (°)	101.6 115.9 142.1<br>90.0, 90.0, 90.0	104.2, 117.4, 140.7<br>90.0, 90.0, 90.0
[a]Resolution (Å)	89.79 – 3.50<br>(3.78 – 3.50)	90.15 – 2.80<br>(2.91 – 2.80)
Unique reflections	21668 (4376)	43000 (4494)
[a]Redundancy	6.2 (6.1)	6.7 (7.0)
[a]Completeness (%)	99.6 (99.5)	99.5 (100.0)
[a] I/σI	5.1 (1.9)	5.5 (1.7)
[a,b]R_{merge}	0.450 (1.942)	0.424 (2.504)
[c]$CC_{1/2}$	0.947 (0.594)	0.949 (0.352)
**Refinement**		
Protein atoms	9366	9933
Ligand molecules	0	2 2OG, 2 Cl
Metal	0	4 Mn
Water	0	312
[d]R_{work}/R_{free}	0.24/0.27	0.25/0.28
B-factors (Å^2)	77.65	47.1
Protein	77.65	47.4
Ligand	-	46.5
Metal	-	44.5
H_2O		38.2
R.m.s. deviation in bond lengths (Å)	0.002	0.019
R.m.s. deviation in bond angles (°)	0.46	1.56
Ramachandran plot (%) favored	99.28	99.4
Ramachandran plot (%) outliers	0.72	0.6
Rotamer outliers (%)	1.5	5.2
PDB ID	9EIR	9EIS

[a]The highest resolution shell is shown in parentheses.
[b]$R_{merge} = \sum_{hkl} \sum_j |I_j(hkl) - \langle I(hkl) \rangle| / \sum_{hkl} \sum_j Ij(hkl)$, where I is the intensity of reflection.
[c]$CC_{1/2}$ is the correlation coefficient of the half datasets
[e]$R_{work} = \sum_{hkl} | |F_{obs}| - |F_{calc}| | / \sum_{hkl} |F_{obs}|$, where F_{obs} and F_{calc} is the observed and the calculated structure factor, respectively. R_{free} is the cross-validation R factor for the test set of reflections (5% of the total) omitted in model refinement.

Table S4. Metal contents of purified and EDTA/dithionite-treated Pd1 and PK2 EFE species as determined by ICP-MS

EFE Sample	Buffer/other	Fe/Subunit	Ni/Subunit
PK2	phosphate	0.042	0.85
Pd1	phosphate	0.052	0.32
PK2	HEPES	0.039	0.86
Pd1	HEPES	0.053	0.23
PK2	HEPES/EDTA/$S_2O_4^{2-}$	0.015	0.026
Pd1	HEPES/EDTA/$S_2O_4^{2-}$	0.041	0.015

Table S5. Ethylene, P5C, and succinate production by PK2 and Pd1 EFEs using varied conditions. The EFE samples (220 nM, 2 mL) were incubated in 25 mM HEPES buffer with 0.5 mM 2OG, 0.5 mM L-Arg, 0.4 mM Fe(II), and 0.6 mM L-ascorbate. Ethylene production was assessed by GC. P5C production was measured by reaction with 2-aminobenzaldehyde. Succinate and the remaining 2OG were quantified by TQ-S triple quadrupole MS/MS and the OPDA assay, respectively. Panels show data for two biological replicates, with similar trends noted for additional experiments.

(A) Varied temperatures ± L-Arg at pH 7.5 for 80 min, quenching by 60 °C for 20 min.

Substance (nmoles)	Temp	L-Arg	PK2	Pd1
Ethylene	25	Yes	793 ± 10	649 ± 51
P5C			122 ± 8	70 ± 7
Succinate			116 ± 21	91 ± 9
Remaining 2OG			45 ± 14	87 ± 42
Ethylene	25	No	-	-
P5C			-	-
Succinate			43 ± 14	59 ± 27
Remaining 2OG			855 ± 71	907 ± 71
Ethylene	37	Yes	742 ± 51	603 ± 44
P5C			147 ± 18	68 ± 13
Succinate			129.4 ± 0.1	101 ± 3
Remaining 2OG			80 ± 42	113 ± 14
Ethylene	37	No		
P5C				
Succinate			47 ± 14	60 ± 21
Remaining 2OG			821 ± 147	916 ± 71

(B) Varied pH and ± L-Arg for 40 min at 22 °C and quenched at 60 °C for 15 min.

Substance (nmoles)	pH	L-Arg	PK2	Pd1
Ethylene	6.5	Yes	800 ± 71	586 ± 22
P5C			175 ± 28	74 ± 9
Succinate			128 ± 21	121 ± 14
Ethylene	6.5	No	-	-
P5C			-	-
Succinate			91 ± 15	121 ± 21
Ethylene	7.5	Yes	666 ± 19	748 ± 35
P5C			191 ± 11	116 ± 19
Succinate			137 ± 8	173 ± 22
Ethylene			-	

P5C	7.5	No	-	
Succinate			68 ± 18	71 ± 12
Ethylene	8	Yes	755 ± 29	719 ± 35
P5C			104 ± 14	89 ± 9
Succinate			102 ± 14	89 ± 22
Ethylene	8	No	-	-
P5C			-	-
Succinate			38 ± 14	45 ± 22

(C) Additional succinate determinations (in nanomoles) using the same reaction conditions.

**Conditions**	**Succinate (nmoles)**
PK2 EFE 25 ºC with Arg	148 ± 24
PK2 EFE 25 ºC no Arg	30 ± 5
PK2 EFE 37 ºC with Arg	141 ± 16
PK2 EFE 37 ºC no Arg	30 ± 9
PK2 EFE pH 6.5 with L-Arg	155 ± 18
PK2 EFE pH 6.5 no L-Arg	100 ± 27
PK2 EFE pH 8 with L-Arg	133 ± 58
PK2 EFE pH 8 no L-Arg	26 ± 2
Pd1 EFE 25 ºC with Arg	104 ± 9
Pd1 EFE 25 ºC no Arg	38 ± 2
Pd1 EFE 37 ºC with Arg	98 ± 7
Pd1 EFE 37 ºC no Arg	40 ± 6
Pd1 EFE pH 6.5 with L-Arg	130 ± 2
Pd1 EFE pH 6.5 no L-Arg	93 ± 18
Pd1 EFE pH 8 with L-Arg	93 ± 28
Pd1 EFE pH 8 no L-Arg	36 ± 8

Table S6. RMSD (Å) of Cα atoms for the four Pd1 EFE chains in the apoprotein and Mn(II)·2OG complex

	Pd1 EFE apoprotein chain A	Pd1 EFE apoprotein chain B	Pd1 EFE apoprotein chain C	Pd1 EFE apoprotein chain D
Pd1 EFE apoprotein chain A	0	0.21	0.29	0.32
Pd1 EFE apoprotein chain B		0	0.31	0.32
Pd1 EFE apoprotein chain C			0	0.19
Pd1 EFE apoprotein chain D				0
Pd1 EFE·Mn·2OG complex chain A	0.38	0.39	0.49	0.45
Pd1 EFE·Mn·2OG complex chain B	0.39	0.35	0.45	0.43
Pd1 EFE·Mn·2OG complex chain C	0.38	0.39	0.41	0.38
Pd1 EFE·Mn·2OG complex chain D	0.54	0.52	0.51	0.52

References

[1] Chaturvedi, S. S., Ramanan, R., Hu, J., Hausinger, R. P., and Christov, C. Z. (2021) Atomic and electronic structure determinants distinguish between ethylene formation and L-arginine hydroxylation reactions mehanisms in the ethylene-forming enzyme, *ACS Catalysis 11*, 1578-1592.
